# Risk of dementia associated with anticholinergic drugs for overactive bladder in adults aged ≥55 years: nested case-control study

**DOI:** 10.1136/bmjmed-2023-000799

**Published:** 2024-11-12

**Authors:** Barbara Iyen, Carol Coupland, Brian Gregory Bell, Darren M Ashcroft, Martin William Orrell, Delia Bishara, Tom Dening, Anthony J Avery

**Affiliations:** 1 Centre for Academic Primary Care, School of Medicine, University of Nottingham, Nottingham, UK; 2 Division of Pharmacy and Optometry, School of Health Sciences, Faculty of Biology, Medicine, and Health, University of Manchester, Manchester, UK; 3 National Institute for Health and Care Research (NIHR) Greater Manchester Patient Safety Research Collaboration (PSRC), University of Manchester, Manchester, UK; 4 Institute of Mental Health, Faculty of Medicine and Health Sciences, University of Nottingham, Nottingham, UK; 5 Institute of Psychiatry, Psychology, and Neuroscience, Department of Psychological Medicine, King's College London, London, UK

**Keywords:** Dementia, Geriatrics, Medicine, Primary health care, Urinary incontinence

## Abstract

**ABSTRACT:**

**Objective:**

To investigate whether different anticholinergic drug treatments for overactive bladder have differential risks for incident dementia, in a large representative population of older adults in England.

**Design:**

Nested case-control study.

**Setting:**

General practices in England providing data to the Clinical Practice Research Datalink (CPRD) GOLD database, with linked patient admission records from secondary care (Hospital Episode Statistics), 1 January 2006 and 16 February 2022

**Participants:**

170 742 patients aged ≥55 years, with a first reported diagnosis of dementia during the study period, matched by age, sex, and general practice with 804 385 individuals without dementia (controls).

**Interventions:**

Cumulative drug use (defined using total standardised daily dose) of different anticholinergic drugs used for the treatment of an overactive bladder, and a non-anticholinergic drug, mirabegron, in the period 3-16 years before a diagnosis of dementia (or equivalent date in matched controls).

**Main outcome measures:**

Odds ratios for onset of dementia associated with the different anticholinergic drugs used for the treatment of an overactive bladder, adjusted for sociodemographic characteristics, clinical comorbidities, and use of other anticholinergic drug treatments.

**Results:**

The study population comprised 62.6% women, and median age was 83 (interquartile range 77-87) years. 15 418 (9.0%) patients with dementia and 63 369 (7.9%) controls without dementia had used anticholinergic drugs for the treatment of an overactive bladder in the 3-16 years before diagnosis (or equivalent date for controls). The adjusted odds ratio for dementia associated with the use of any anticholinergic drug used to treat an overactive bladder was 1.18 (95% confidence interval (CI) 1.16 to 1.20), and was higher in men (1.22, 1.18 to 1.26) than women (1.16, 1.13 to 1.19). The risk of dementia was substantially increased with the use of oxybutynin hydrochloride (adjusted odds ratio 1.31, 95% CI 1.21 to 1.42 and 1.28, 1.15 to 1.43 for use of 366-1095 and >1095 total standardised daily doses, respectively), solifenacin succinate (1.18, 1.09 to 1.27 and 1.29, 1.19 to 1.39), and tolterodine tartrate (1.27, 1.19 to 1.37 and 1.25, 1.17 to 1.34). No significant increases in the risk of dementia associated with darifenacin, fesoterodine fumarate, flavoxate hydrochloride, propiverine hydrochloride, and trospium chloride were found. The association between mirabegron, a non-anticholinergic drug, and dementia was variable across the dose categories and might be caused by previous use of anticholinergic drugs for the treatment of an overactive bladder in these individuals.

**Conclusions:**

Of the different anticholinergic drugs used to treat an overactive bladder, oxybutynin hydrochloride, solifenacin succinate, and tolterodine tartrate were found to be most strongly associated with the risk of dementia in older adults. This finding emphasises the need for clinicians to take into account the possible long term risks and consequences of the available treatment options for an overactive bladder in older adults, and to consider prescribing alternative treatments that might be associated with a lower risk of dementia.

**WHAT IS ALREADY KNOWN ON THE TOPIC**

- Anticholinergic drugs used in managing the symptoms of an overactive bladder in older adults have been linked to a risk of dementia, but variations in risk between different anticholinergic drugs are not known

**WHAT THIS STUDY ADDS**

- In this nested case-control study of 170 742 patients with dementia and 804 385 matched controls, the risk of dementia was found to be substantially associated with cumulative use of oxybutynin hydrochloride, solifenacin succinate, and tolterodine tartrate
- No significant increases were found in the risk of dementia associated with darifenacin, fesoterodine fumarate, flavoxate hydrochloride, propiverine hydrochloride, and trospium chloride

**HOW THIS STUDY MIGHT AFFECT RESEARCH, PRACTICE, OR POLICY**

- These findings of an increased risk of dementia with some anticholinergic drugs used to treat an overactive bladder suggest the need for clinicians to consider prescribing alternative treatments that are not significantly associated with a risk of dementia

## Introduction

 Prolonged treatment with anticholinergic drugs has been linked to long term cognitive decline and dementia,^1^,^3^ and is a potentially modifiable risk factor. Several studies^4 5^ have found a strong longitudinal association between the use of anticholinergic drugs used to treat bladder symptoms in older adults and the risk of dementia. Despite this finding, use of these drugs nearly doubled in older adults in England over the past 20 years.^6^

Overactive bladder, a chronic condition characterised by urinary symptoms of urgency, with or without urinary incontinence, frequency, and nocturia, is a common clinical presentation among elderly men and women. In treating an overactive bladder, guidelines from the National Institute for Health and Care Excellence (NICE),^7 8^ as well as local area prescribing committees,^9 10^ state that anticholinergic drugs for an overactive bladder have similar efficacy and side effect profiles, and hence recommend that clinicians should prescribe anticholinergic drugs with the lowest acquisition cost, such as oxybutynin hydrochloride, tolterodine tartrate, and darifenacin.

Although anticholinergic drugs used in managing an overactive bladder have been associated with a risk of dementia as a group, individual drug treatments vary in their ability to cross the blood-brain barrier under normal conditions.^11 12^ Consequently, some anticholinergic drugs might pose a higher risk than others. For example, oxybutynin hydrochloride, a small molecule, readily crosses the blood-brain barrier and blocks M1 receptors.^11^ This action could contribute to the formation of neurofibrillary tangles and amyloid plaques associated with dementia,^13^ although evidence of the possible association between muscarinic blockade and amyloid plaques and neurofibrillary tangles is mixed.^14^,^16^ Also, larger doses of oxybutynin hydrochloride have been linked to greater memory problems.^4 11^ In contrast, a few clinical trials of short duration that have assessed the risk of cognitive decline associated with the use of solifenacin succinate^17^ and darifenacin^11^ in older adults have shown no impairment of memory or other cognitive functions.

As well as the use of anticholinergic drugs, an overactive bladder can be effectively managed with mirabegron, a β3 adrenoceptor agonist. This drug works by inducing relaxation of the detrusor smooth muscle, suppressing detrusor overactivity, and enhancing bladder capacity.^18^ NICE guidelines, however, only recommend mirabegron as an alternative treatment when anticholinergic drugs are contraindicated or clinically ineffective.^19^

Despite the emerging evidence suggesting that some anticholinergic drugs used for the treatment of an overactive bladder are safer than others in relation to the risk of dementia, so far this limited evidence has not been adopted into clinical guidelines, and general practitioners continue to prescribe drugs, such as oxybutynin hydrochloride, that might be high risk. Hence in this study, we investigated the specific risk of dementia associated with different anticholinergic drugs, as well as mirabegron, used to treat an overactive bladder, in a large representative population of older individuals in England.

## Methods

### Study design and data source

We conducted a nested case-control study with electronic health records of patients registered with general practices contributing to the Clinical Practice Research Datalink (CPRD) GOLD database. CPRD GOLD is a database of routine primary care electronic healthcare records of patients in the UK.^20^ The database has data on personal characteristics, symptoms, tests, diagnoses, treatments, health related behaviours, and referrals to secondary care. CPRD GOLD covers about 4.5% of the UK population, and patients are broadly representative of the UK general population in terms of age, sex, and ethnic group.^20^ Records for this study were available from 954 general practices that contributed to the database during the study period from 1 January 2006 to 16 February 2022.

Patients' primary care records from CPRD GOLD were linked to their secondary care records from Hospital Episode Statistics and to records of socioeconomic status. The measure of socioeconomic status was the English index of multiple deprivation 2019 for patients, an area level weighted composite measure derived from different indicators covering domains of material deprivation: housing, employment, income, access to services, education and skills, crime, and living environment.^21^ The index of multiple deprivation classifies areas into five groups based on relative deprivation, with group 1 being the least deprived and group 5 being the most deprived.

### Study population

**Figure 1:**
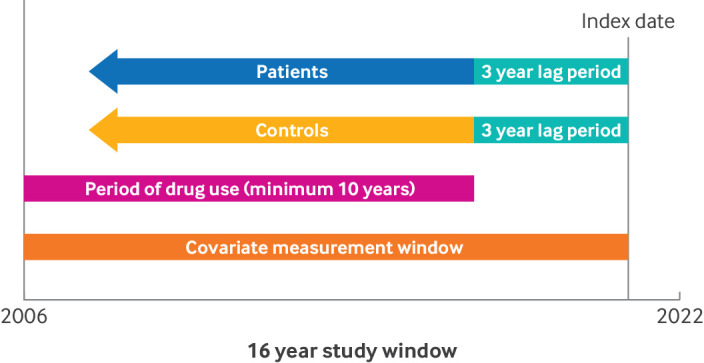
Case-control study design, showing the study follow-up period, and time lines for drug use and assessment of covariates

We included patients aged ≥55 years who had a recorded primary care or secondary care diagnosis of dementia (identified with validated Read or ICD-10 (international classification of diseases, 10th revision) codes for various types of dementia but not including codes for mild cognitive impairment) or primary care records of prescriptions for drugs used specifically to treat dementia (acetylcholinesterase inhibitors or memantine hydrochloride), or both. Each patient with dementia was matched with up to five individuals without dementia (controls), by age, sex, general practice, and calendar time (same time period of follow-up) with incidence density sampling and allowing for replacement of controls. The index date was defined as the earliest recorded date of a diagnosis of dementia (or date of the first prescription of a drug treatment for dementia) for patients, whereas controls were assigned the date of the diagnosis of dementia of their matched patient. Patients and controls were included only if they had at least 13 years of electronic health data recorded before the index date, so that we could assess the use of anticholinergic treatments for an overactive bladder over a minimum period of 10 years (excluding the three year period before the index date) (figure 1).

### Independent variables

The primary independent variable was total cumulative use of anticholinergic drugs used to treat an overactive bladder: darifenacin, fesoterodine fumarate, flavoxate hydrochloride, oxybutynin hydrochloride, propiverine hydrochloride, solifenacin succinate, tolterodine tartrate, and trospium chloride. Total cumulative use of a non-anticholinergic drug for an overactive bladder, mirabegron, was included for comparison. In accordance with methods used in previous research of anticholinergic drug treatment and risk of dementia,^5^ all prescriptions in the last three years of follow-up before the index date (a diagnosis of dementia or equivalent in matched controls) were excluded to reduce protopathic bias because drugs might have been prescribed for an overactive bladder in the prodromal period before a diagnosis of dementia.

Total standardised daily dose was used as the measure of patients' total cumulative use of each of the anticholinergic drugs used to treat an overactive bladder, based on the method of Gray et al^1^ and Coupland et al.^5^ Total standardised daily dose has been used in numerous research studies to determine the risk of clinical outcomes associated with standardised use of drugs such as benzodiazepines,^22 23^ combined central nervous system drug treatments,^24^ as well as anticholinergic drugs.^1 5^ The use of total standardised daily dose allowed us to quantify and standardise patients' cumulative use of different doses of various drug treatments during the study period, with standardised conversion measures derived from the World Health Organization's defined daily dose^25^ (online supplemental table S1), which is the assumed average maintenance dose per day for a drug used for its main indication in adults. Total standardised daily dose was derived using the following steps: (a) standardised daily dose=prescribed daily dose (mg) of drug treatment divided by WHO defined daily dose; (b) standardised use=standardised daily dose (for each type of drug used for an overactive bladder and for each patient) multiplied by the length of treatment (cumulative use (in days) of the drug used for an overactive bladder during the study period); and (c) total standardised daily dose=sum of standardised use for each drug for each patient, during the study period.

### Covariates

We adjusted for patients' sociodemographic characteristics, clinical comorbidities, variables known to increase the risk of dementia,^26^,^29^ and use of other anticholinergic drugs. These covariates included non-modifiable risk factors, such as ethnic group and patient level of deprivation, and clinical comorbidities and variables that increase the risk of dementia,^26^,^29^ such as: smoking status, alcohol consumption, body mass index, anxiety, asthma, atrial fibrillation, bipolar disorder, chronic kidney disease, chronic obstructive pulmonary disease, coronary heart disease, depression, Down's syndrome, head injury, heart failure, hyperlipidaemia, hypertension, learning disability, schizophrenia, stroke, subarachnoid haemorrhage, transient ischaemic attack, type 1 diabetes, and type 2 diabetes. We identified all records of clinical comorbidities within the study period until the index date, and used the most recent records of alcohol consumption and smoking status before the index date as confounders in the study.

We also accounted for patients' use of anticholinergic drugs other than those used to treat an overactive bladder, prescribed anytime during the study period up to three years before the index date. These included drugs identified from previous studies,^30^ the British National Formulary, and further discussion with the study team of clinicians and academics with experience of research of anticholinergic drugs (online supplemental table S2). The anticholinergic effect on cognition score classifies anticholinergic drugs, with a scale of 0-3 based on their known central anticholinergic burden and likelihood of being associated with cognitive impairment.^31^ The anticholinergic effect on cognition score of all anticholinergic drugs used to treat patients with conditions other than an overactive bladder in the 13 year period before a dementia diagnosis (or index date) was calculated to derive a mean score, and these mean scores were included as confounders in the multivariable analyses. All analyses were grouped on the matched sets and therefore controlled for any confounding effect of age, sex, and general practice.

### Statistical analyses

Baseline personal and clinical characteristics were determined for all patients with dementia and their matched controls, and presented as absolute numbers (percentages), mean (standard deviation, SD), and median (interquartile range, IQR) for categorical, continuous normally distributed, and continuous non-normally distributed variables, respectively. Pearson's χ^2^ test was used to assess differences in categorical clinical variables between the groups.

Missing values for body mass index were estimated with multiple imputations by chained equations based on all of the other available patient variables and outcome (dementia) to generate 10 imputed datasets.^32^,^34^ Imputed datasets were pooled into one dataset with Rubin's rules.^35^ Missing categorical personal variables, such as ethnic group, smoking status, and alcohol consumption, were coded as separate unknown or missing categories; assumptions were made that individuals with missing or unrecorded clinical variables or comorbidities did not have the condition.

Anticholinergic drug use for the treatment of an overactive bladder in the 3-16 year period before a diagnosis of dementia was categorised into five categories^1 5^ (no drug used, 1-90, 91-365, 366-1095, and >1095 total standardised daily doses).^1 5^ Conditional logistic regression estimated the odds ratios for dementia associated with different anticholinergic drugs used to treat an overactive bladder and mirabegron, after adjusting for confounding variables. In clinical practice, patients treated for an overactive bladder often take multiple anticholinergic drugs, and therefore we also accounted for use of other commonly prescribed anticholinergic drugs for an overactive bladder in our multivariable conditional logistic regression analyses. To ensure that the study results were robust and generalisable, subgroup analyses were done to assess associations between dementia and anticholinergic drugs used for the treatment of an overactive bladder in: adults aged <80 years and ≥aged 80 years at the time of diagnosis of dementia (or the index date); men and women separately; and subgroups of patients prescribed mirabegron who had not previously used any anticholinergic drug for the treatment of an overactive bladder. All analyses were conducted with Stata SE version 17 statistical package, and significance was defined as P≤0.05. The study was reported in line with the reporting of studies conducted using observational routinely collected health data statement for pharmacoepidemiology (RECORD-PE) guidelines.

### Patient and public involvement

The design of this study drew on feedback from public engagement after the anticholinergic drugs and dementia study by Coupland et al.^5^ The design was further discussed with our study patient and public involvement lead to ensure relevance to the general public and particularly to older adults who have a higher risk of developing dementia. The study patient and public involvement lead, who has acted as a carer of a person with dementia, reviewed the study protocol lay summary, discussed study findings, and will be involved in the dissemination of the findings of the study. The results of the study will also be disseminated to other relevant patient and public groups.

## Results

From the CPRD GOLD base population of 3 575 273 individuals, we identified 170 773 patients with dementia. Thirty one^31^ of these patients had no eligible matched controls and were therefore excluded. The final study population comprised 170 742 patients and 804 385 matched controls, identified from 954 general practices contributing data to CPRD GOLD from 1 January 2006 to 16 February 2022. Median age of the patients was 83 years (IQR 77-87) and 62.6% were women. Data for sex were taken from information in CPRD GOLD rather than from patient reported gender. Records of ethnic group were available for 42.4% of patients in the study, and 95.1% of those with ethnic group records were white (table 1). Among the overall study population, 175 942 (17.8%) had missing records for body mass index before the index date, and these were imputed with multiple imputation techniques, as previously described. Online supplemental figure S1 shows a plot of the distribution of observed and imputed body mass index records. Mean body mass index at baseline was slightly lower in patients (25.7 (SD 4.5)) than controls (26.5 (4.7)). The prevalence of alcohol consumption (current alcohol drinking status) was lower in patients (47.7%) than controls (57.8%) but the prevalence of current smoking was marginally higher at baseline in patients (7.8%) than controls (7.6%).

**Table 1:** Baseline personal characteristics of patients with dementia and non-dementia controls (n=975 127)

Characteristics*	Patients (n=170 742)	Controls (n=804 385)
Mean (SD) age (years) at diagnosis or index date	82.3 (7.7)	81.8 (7.6)
Median (IQR) age (years) at diagnosis or index date	83 (78-88)	83 (77-87)
Age (years) at diagnosis or index date:		
55-64	4309 (2.5)	21 524 (2.7)
65-74	21 649 (12.7)	107 702 (13.4)
75-84	72 521 (42.5)	355 477 (44.2)
85-94	66 493 (38.9)	300 824 (37.4)
≥95	5770 (3.4)	18 858 (2.3)
Sex:		
Men	63 865 (37.4)	299 348 (37.2)
Women	106 877 (62.6)	505 037 (62.8)
No of patients with ethnic group records†:	78 071 (100)	348,316 (100)
White	74 641 (95.6)	330 750 (95.0)
Bangladeshi	44 (0.06)	149 (0.04)
Black African	105 (0.13)	383 (0.11)
Black Caribbean	428 (0.55)	1366 (0.39)
Black other	69 (0.09)	227 (0.07)
Chinese	61 (0.08)	381 (0.11)
Indian	408 (0.52)	2022 (0.58)
Mixed	127 (0.16)	546 (0.16)
Other Asian	132 (0.17)	718 (0.21)
Pakistani	157 (0.20)	753 (0.22)
Other	1899 (2.43)	11 021 (3.17)
Index of multiple deprivation (group):		
1 (least deprived)	18 294 (10.7)	91 237 (11.3)
2	17 195 (10.1)	85 780 (10.7)
3	18 068 (10.6)	80 994 (10.1)
4	13 220 (7.7)	59 933 (7.5)
5 (most deprived)	12 290 (7.2)	50 153 (6.2)
Missing index of multiple deprivation records	91 675 (53.7)	436 288 (54.2)
Body mass index:		
Mean (SD) body mass index	25.7 (4.5)	26.5 (4.7)
Missing body mass index records	42 634 (25.0)	133 308 (16.6)
Smoking status (most recent before index date):		
Non-smoker	80 020 (46.9)	414 421 (51.5)
Ex-smoker	47 783 (28.0)	244 276 (30.4)
Current smoker	13 247 (7.8)	61 262 (7.6)
Missing smoking records	29 692 (17.4)	84 426 (10.5)
Alcohol status (most recent before index date):		
Non-alcohol drinker	39 280 (23.0)	176 261 (21.9)
Ex-alcohol drinker	6775 (4.0)	24 230 (3.0)
Alcohol drinker	81 414 (47.7)	464 751 (57.8)
Missing alcohol records	43 273 (25.3)	139 143 (17.3)

Data are number (%) unless indicated otherwise.

* Baseline characteristics measured at index date.

† Records of ethnic group were available for 426 387 (42.4%) individuals in the study who were eligible for linkage with Hospital Episodes Statistics. Therefore, records were missing for 92 671 (54.3%) patients and 456 069 (56.7%) controls.

IQR, interquartile range; SD, standard deviation.

Table 2 shows the prevalence of comorbidities in patients and controls, up to three years before the index date. Patients had a higher prevalence of mental health related conditions, such as anxiety, bipolar disorder, depression, learning disabilities, Down's syndrome, and schizophrenia, and head injury. Other conditions, such as atrial fibrillation, chronic kidney disease, chronic obstructive pulmonary disease, coronary heart disease, heart failure, stroke, transient ischaemic attack, subarachnoid haemorrhage, and type 1 and type 2 diabetes, were also more prevalent in patients than controls. Asthma, hyperlipidaemia, and hypertension were more prevalent in controls. Table 2 also shows the use of anticholinergic drugs for conditions other than an overactive bladder in the 3-16 years before the index date. Although patients had a marginally higher prevalence of prescriptions of these other anticholinergic drugs, more anticholinergic drugs with an anticholinergic effect on cognition score of 0 or 1 were prescribed in these patients than controls.

**Table 2:** Clinical characteristics at baseline for patients with dementia and non-dementia controls

Clinical characteristics	Patients (n=170 742)	Controls (n=804 385)	P value*
Comorbidities:			
Anxiety	27 768 (16.3)	106 742 (13.3)	<0.001
Asthma	17 920 (10.5)	88 575 (11.0)	<0.001
Atrial fibrillation	26 281 (15.4)	102 043 (12.7)	<0.001
Bipolar disorder	1132 (0.7)	2199 (0.3)	<0.001
Chronic kidney disease	42 929 (25.1)	196 319 (24.4)	<0.001
Chronic obstructive pulmonary disease	14 083 (8.3)	64 661 (8.0)	0.004
Coronary heart disease	36 518 (21.4)	158 491 (19.7)	<0.001
Depression	38 658 (22.6)	130 598 (16.2)	<0.001
Down's syndrome	319 (0.2)	34 (0.0)	<0.001
Head injury	7042 (4.1)	22 425 (2.8)	<0.001
Heart failure	12 788 (7.5)	55 343 (6.9)	<0.001
Hyperlipidaemia	28 839 (16.9)	137 942 (17.2)	0.010
Hypertension	96 161 (56.3)	470 911 (58.5)	<0.001
Learning disabilities	896 (0.5)	1380 (0.2)	<0.001
Schizophrenia	2405 (1.4)	4412 (0.6)	<0.001
Stroke	24 026 (14.1)	70 777 (8.8)	<0.001
Subarachnoid haemorrhage	949 (0.6)	2561 (0.3)	<0.001
Transient ischaemic attack	14 899 (8.7)	49 514 (6.2)	<0.001
Type 1 diabetes	1375 (0.8)	4618 (0.6)	<0.001
Type 2 diabetes	28 167 (16.5)	115 905 (14.4)	<0.001
Use of other anticholinergic drugs†	99 969 (58.6)	467 774 (58.2)	0.003
Anticholinergic effect on cognition score (n=98 339 patients and n=457 808 controls)‡:			
Score 0	39 634 (40.3)	178 984 (39.1)	<0.001
Score 1	23 543 (23.9)	99 251 (21.7)	
Score 2	26 964 (27.4)	131 294 (28.7)	
Score 3	8198 (8.3)	48 279 (10.6)	

Data are number (%).

* Tests of significance for categorical variables were derived from Pearson's χ2 test.

† Use of anticholinergic drugs for conditions other than an overactive bladder in the 3-16 year period before the index date.

‡ Mean anticholinergic effect on cognition score of anticholinergic drugs for conditions other than an overactive bladder used in the 16 years before the index date.

### Use of anticholinergic drugs for overactive bladder

Anticholinergic drugs for an overactive bladder were prescribed for 78 787 (8.1%) of the 975 127 individuals in the study: 15 418 (9.0%) patients and 63 369 (7.9%) controls. Table 3 shows that the drugs most commonly prescribed in patients and controls were oxybutynin hydrochloride (4.7% of patients and 4.1% of controls), tolterodine tartrate (4.1% and 3.5% of patients and controls, respectively), and solifenacin succinate (2.8% and 2.4% of patients and controls, respectively). Online supplemental table S3 shows the baseline characteristics of patients prescribed the different drugs for an overactive bladder.

**Table 3:** Prescribing of anticholinergic drugs and mirabegron for overactive bladder in patients with dementia and non-dementia controls, in the 3-16 year period before a diagnosis of dementia or index date

Anticholinergic drug	Patients (n=170 742)		Controls (n=804 385)	
	No (%) prescribed drug treatment	Cumulative use of drug treatment* (median (IQR))	No (%) prescribed drug treatment	Cumulative use of drug treatment* (median (IQR))
Darifenacin	66 (0.04)	84 (28-308)	266 (0.03)	112 (28-392)
Fesoterodine fumarate	528 (0.3)	140 (46.5-560)	2118 (0.3)	112 (28-448)
Flavoxate hydrochloride	493 (0.3)	22.5 (21-84)	2106 (0.3)	22.5 (21-67.5)
Oxybutynin hydrochloride	7952 (4.7)	37.3 (14-186.7)	33 579 (4.1)	28.7 (10-136)
Propiverine hydrochloride	468 (0.3)	84 (28-492)	1725 (0.2)	56 (28-392)
Solifenacin succinate	4778 (2.8)	202 (58-812)	19 058 (2.4)	168 (56-666)
Tolterodine tartrate	7060 (4.1)	112 (28-588)	28 180 (3.5)	98 (28-476)
Trospium chloride	1518 (0.9)	90 (30-367.5)	5802 (0.7)	84 (30-282)
Mirabegron (non-anticholinergic drug)	416 (0.2)	150 (60-439)	1410 (0.2)	120 (30-343)

* Total (cumulative) standardised daily dose of prescribed drug treatment (standardised doses derived from World Health Organization's defined daily dose for different drug treatments).

IQR, interquartile range.

Table 4 shows the unadjusted and adjusted odds ratios of dementia associated with the use of any anticholinergic drug as well as individual anticholinergic drugs and mirabegron (non-anticholinergic drug) to treat an overactive bladder, between three and 16 years before the index date. The adjusted odds ratio for dementia associated with the use of any anticholinergic drug for an overactive bladder compared with no use of these drug treatments was 1.18 (95% CI 1.16 to 1.20). Among the different anticholinergic drugs used to treat an overactive bladder, we found considerable dose-response increases for the association between dementia and increasing dose levels of oxybutynin hydrochloride (adjusted odds ratio 1.04, 95% CI 1.01 to 1.07, for 1-90 total standardised daily doses; 1.28, 1.15 to 1.43, for >1095 total standardised daily doses), solifenacin succinate (1.02, 0.97 to 1.08; 1.29, 1.19 to 1.39), and tolterodine tartrate (1.05, 1.01 to 1.09; 1.25, 1.17 to 1.34). We found no substantial increase in the risk of dementia associated with the use of other anticholinergics drugs (ie, darifenacin, fesoterodine fumarate, flavoxate hydrochloride, propiverine hydrochloride, and trospium chloride).

The risk of dementia associated with mirabegron was variable across different dose levels, with an increase only with mirabegron 91-365 total standardised daily doses and 366-1095 total standardised daily doses. This finding might be because 86.2% of individuals prescribed mirabegron (1574 of 1826 patients) had previously used anticholinergic drugs for an overactive bladder. Also, the number of individuals in the >1095 total standardised daily dose subgroup was small, resulting in insufficient statistical power to detect precise measures of effect. Of the 78 787 individuals who used anticholinergic drugs, 77% (60 743) had previously used at least one different anticholinergic drug for treatment of an overactive bladder.

**Table 4:** Crude and adjusted odds ratios for dementia by total cumulative use of different anticholinergic drugs and mirabegron for an overactive bladder in patients with dementia and non-dementia controls, 3-16 years before a diagnosis of dementia*

Drug treatment and cumulative use of anticholinergic drug (total standardised daily dose)	No (%) of patients (n=170 742)	No (%) of controls (n=804 385)	Unadjusted odds ratio (95% CI)	Adjusted odds ratio† (95% CI)
Any anticholinergic drug treatment for overactive bladder:				
No use of anticholinergic drugs to treat overactive bladder	155 324 (90.97)	741 016 (92.12)	1.00 (Reference)	1.00 (Reference)
Use of anticholinergic drugs for overactive bladder	15 418 (9.03)	63 369 (7.88)	1.15 (1.13 to 1.17)	1.18 (1.16 to 1.20)
Darifenacin:				
None	170 676 (99.96)	804 119 (99.97)	1.00 (Reference)	1.00 (Reference)
1-90	36 (0.02)	127 (0.02)	1.34 (0.92 to 1.95)	1.08 (0.74 to 1.58)
91-365	15 (0.01)	70 (0.01)	0.99 (0.56 to 1.73)	0.86 (0.49 to 1.53)
366-1095	10 (0.01)	51 (0.01)	0.83 (0.41 to 1.67)	0.79 (0.39 to 1.59)
>1095	5 (0.00)	18 (0.00)	1.39 (0.52 to 3.75)	1.26 (0.46 to 3.50)
Fesoterodine fumarate:				
None	170 214 (99.69)	802 267 (99.74)	1.00 (Reference)	1.00 (Reference)
1-90	220 (0.13)	997 (0.12)	1.02 (0.88 to 1.18)	0.91 (0.78 to 1.05)
91-365	138 (0.08)	530 (0.07)	1.24 (1.03 to 1.50)	1.08 (0.89 to 1.31)
366-1095	99 (0.06)	349 (0.04)	1.30 (1.04 to 1.63)	1.09 (0.87 to 1.38)
>1095	71 (0.04)	242 (0.03)	1.36 (1.04 to 1.77)	1.15 (0.87 to 1.51)
Flavoxate hydrochloride:				
None	170 249 (99.71)	802 279 (99.74)	1.00 (Reference)	1.00 (Reference)
1-90	379 (0.22)	1675 (0.21)	1.04 (0.93 to 1.17)	0.90 (0.81 to 1.01)
91-365	62 (0.04)	256 (0.03)	1.13 (0.86 to 1.50)	0.97 (0.73 to 1.28)
366-1095	32 (0.02)	130 (0.02)	1.15 (0.77 to 1.69)	1.07 (0.72 to 1.59)
>1095	20 (0.01)	45 (0.01)	2.13 (1.25 to 3.61)	1.71 (1.00 to 2.96)
Oxybutynin hydrochloride:				
None	162 790 (95.34)	771 806 (95.95)	1.00 (Reference)	1.00 (Reference)
1-90	5210 (3.05)	22 666 (2.82)	1.08 (1.04 to 1.11)	1.04 (1.01 to 1.07)
91-365	1393 (0.82)	5361 (0.67)	1.21 (1.14 to 1.28)	1.14 (1.07 to 1.21)
366-1095	901 (0.53)	3031 (0.38)	1.39 (1.29 to 1.50)	1.31 (1.21 to 1.42)
>1095	448 (0.26)	1521 (0.19)	1.39 (1.25 to 1.55)	1.28 (1.15 to 1.43)
Propiverine hydrochloride:				
None	170 274 (99.73)	802 660 (99.79)	1.00 (Reference)	1.00 (Reference)
1-90	241 (0.14)	974 (0.12)	1.16 (1.01 to 1.34)	1.02 (0.88 to 1.18)
91-365	92 (0.05)	305 (0.04)	1.40 (1.11 to 1.77)	1.17 (0.92 to 1.49)
366-1095	76 (0.04)	246 (0.03)	1.47 (1.13 to 1.90)	1.17 (0.90 to 1.53)
>1095	59 (0.03)	200 (0.02)	1.39 (1.04 to 1.86)	1.18 (0.87 to 1.59)
Solifenacin succinate:				
None	165 964 (97.20)	785 327 (97.63)	1.00 (Reference)	1.00 (Reference)
1-90	1789 (1.05)	7910 (0.98)	1.06 (1.01 to 1.12)	1.02 (0.97 to 1.08)
91-365	1109 (0.65)	4444 (0.55)	1.17 (1.10 to 1.25)	1.11 (1.04 to 1.19)
366-1095	952 (0.56)	3558 (0.44)	1.25 (1.16 to 1.34)	1.18 (1.09 to 1.27)
>1095	928 (0.54)	3146 (0.39)	1.39 (1.29 to 1.49)	1.29 (1.19 to 1.39)
Tolterodine tartrate:				
None	163 682 (95.87)	776 205 (96.50)	1.00 (Reference)	1.00 (Reference)
1-90	3236 (1.90)	14 051 (1.75)	1.08 (1.04 to 1.12)	1.05 (1.01 to 1.09)
91-365	1565 (0.92)	6066 (0.75)	1.21 (1.15 to 1.28)	1.15 (1.08 to 1.22)
366-1095	1153 (0.68)	4054 (0.50)	1.34 (1.25 to 1.43)	1.27 (1.19 to 1.37)
>1095	1106 (0.65)	4009 (0.50)	1.29 (1.21 to 1.38)	1.25 (1.17 to 1.34)
Trospium chloride:				
None	169 224 (99.11)	798 583 (99.28)	1.00 (Reference)	1.00 (Reference)
1-90	781 (0.46)	3292 (0.41)	1.12 (1.04 to 1.21)	0.99 (0.91 to 1.07)
91-365	356 (0.21)	1276 (0.16)	1.31 (1.16 to 1.47)	1.12 (0.99 to 1.27)
366-1095	244 (0.14)	735 (0.09)	1.56 (1.35 to 1.80)	1.31 (1.13 to 1.52)
>1095	137 (0.08)	499 (0.06)	1.27 (1.05 to 1.54)	1.12 (0.92 to 1.37)
Mirabegron (non-anticholinergic drug):				
None	170 326 (99.76)	802 975 (99.82)	1.00 (Reference)	1.00 (Reference)
1-90	152 (0.09)	647 (0.08)	1.08 (0.91 to 1.30)	0.91 (0.76 to 1.10)
91-365	135 (0.08)	420 (0.05)	1.46 (1.20 to 1.78)	1.27 (1.04 to 1.56)
366-1095	116 (0.07)	288 (0.04)	1.90 (1.53 to 2.36)	1.62 (1.30 to 2.03)
>1095	13 (0.01)	55 (0.01)	1.10 (0.60 to 2.03)	0.89 (0.48 to 1.66)

* Dementia outcome includes all dementia subtypes.

† Multivariable model adjusted for ethnic group, patient level of deprivation, smoking status, alcohol consumption, body mass index, records of anxiety, asthma, atrial fibrillation, bipolar disorder, chronic kidney disease, chronic obstructive pulmonary disease, coronary heart disease, depression, Down's syndrome, head injury, heart failure, hyperlipidaemia, hypertension, learning disability, schizophrenia, stroke, subarachnoid haemorrhage, transient ischaemic attack, type 1 diabetes, type 2 diabetes, mean anticholinergic effect of cognition score of other prescribed anticholinergic drugs for conditions other than an overactive bladder, prescription of other commonly prescribed anticholinergic drugs for an overactive bladder (oxybutynin hydrochloride, solifenacin succinate, and tolterodine tartrate); with case-control matching by age, sex, and general practice.

CI, confidence interval.

In our sensitivity analysis, we assessed the association between anticholinergic drugs for the treatment of an overactive bladder and risk of dementia in adults aged <80 years and aged ≥80 years at the time they received a diagnosis of dementia or at the index date. Findings in both age groups were similar to the main analyses but associations between anticholinergic agents (oxybutynin hydrochloride, solifenacin succinate, and tolterodine tartrate) and increased risk of dementia were stronger in adults aged <80 years than in those aged ≥80 years (online supplemental table S4a and table S4b). In separate subgroup analyses in men and women, the odds ratio for dementia associated with the use of any anticholinergic drug was higher in men (adjusted odds ratio 1.22, 95% CI 1.18 to 1.26) than in women (1.16, 1.13 to 1.19). Associations were similar in men and women for oxybutynin hydrochloride, but slightly stronger in men than women for solifenacin succinate and tolterodine tartrate. Similar to the main analyses, effect sizes for mirabegron were variables across dose levels with no clear difference between men and women (online supplemental table S5).

Lastly, sensitivity analyses were also done to assess associations with dementia in the subgroup of patients who were prescribed mirabegron and had no recorded use of anticholinergic drugs for the treatment of an overactive bladder. A total of 1574 of 1826 patients with records of treatment with mirabegron were excluded, and therefore the analyses included 252 patients who received mirabegron in a population of 973 534 patients comprising 170 376 patients and 803 158 controls (a further 19 patients in the study population were also excluded because all of the other patients in their matched set were among the 1574 excluded patients). Because of the small numbers of patients who were treated with mirabegron, this subgroup analysis lacked sufficient power to detect precise estimates of measures of effect, as shown in the wide 95% CIs (online supplemental table S6).

## Discussion

### Principal findings

In this large population based, nested case-control study, the risk of dementia increased substantially with greater cumulative use of the anticholinergic drugs oxybutynin hydrochloride, solifenacin succinate, and tolterodine tartrate in the 3-16 year period before diagnosis. These associations were stronger in those aged <80 years at the time of diagnosis than those aged ≥80 years. We found a higher risk of dementia associated with the use of anticholinergic drugs for an overactive bladder in men than in women. Although similar associations with dementia were seen in men and women for oxybutynin hydrochloride, slightly stronger associations were found in men than women for solifenacin succinate and tolterodine tartrate. No significant increase in the risk of dementia was found with cumulative use of other anticholinergic drugs for an overactive bladder (ie, darifenacin, fesoterodine fumarate, flavoxate hydrochloride, propiverine hydrochloride, and trospium chloride). The risk of dementia varied with different dose levels of mirabegron, a non-anticholinergic drug for the treatment of an overactive bladder, and thisfinding could be attributed to previous use of anticholinergic drugs as well as the small number of individuals in some of the treatment dose categories.

### Strengths and weaknesses of this study

This large population based study assessed the risk of dementia associated with long term use of different treatments for an overactive bladder routinely prescribed in clinical practice. Patients in the CPRD GOLD database are broadly representative of the UK general population in terms of age, sex, and ethnic group,^20^ and therefore the findings of this study are generalisable to the general population of older adults in general practice. Because patients' primary care records were linked to their secondary care records in Hospital Episode Statistics, we could determine a diagnosis of dementia from both settings.

Some limitations are inherently associated with the use of electronic health records.^36^ One limitation is the misclassification of outcomes because of non-recording or misdiagnosis. Of particular concern is the possibility of undiagnosed dementia or early cognitive impairment in individuals in the control group. Although we tried to minimise this risk by inclusion of patients with records of prescriptions for drugs only used to treat dementia (acetylcholinesterase inhibitors or memantine hydrochloride), a residual risk of misclassification exists that would reduce the estimated effects towards the null. Also, records of use of over-the-counter drug treatments are not available in electronic health records. Hence the potential for residual confounding exists from patients' use of over-the-counter drug treatments, such as antihistamines, which have some anticholinergic properties but could not be accounted for in the study analyses. A differential effect of this confounding between the patient and control groups is, however, unlikely.

Our study also assessed the risk of dementia associated with the use of a non-anticholinergic drug, mirabegron. We found that 86.2% of patients prescribed mirabegron had previously been treated with an anticholinergic agent for an overactive bladder. Mirabegron is rarely prescribed as a first line treatment for an overactive bladder in UK clinical practice, and hence our study finding of an increase in the risk of dementia with some dose levels of mirabegron might be because of unmeasured confounding from previous use of anticholinergic drugs. Confounding by indication is also possible, where patients with symptoms of an overactive bladder are given first line treatment with mirabegron instead of an anticholinergic drug when they are already thought to be at risk of cognitive impairment.

Reverse causality is a key limitation of case-control studies, but protopathic bias was minimised in our study by excluding from our analyses, anticholinergic drugs prescribed for an overactive bladder in the three years before a diagnosis of diagnosis, because these drugs might have been prescribed for symptoms in the prodromal period before a diagnosis of dementia.^37^ In terms of the quality of the initial data capture, primary care databases are largely dependent on the judgment of clinicians and patient understanding, especially for self-reported variables, such as smoking and alcohol consumption. Also, variables such as body mass index might not be measured consistently for all patients. We took steps to deal with missing data in our analyses, but all of the variables of interest are representative of the quality of information that is obtained or available in a typical clinical consultation. Thus our study findings reflect a pragmatic evaluation of the risk of dementia associated with the use of anticholinergic drugs for the treatment of an overactive bladder in primary care.

### Comparison with other studies

Although many studies have shown an association between anticholinergic drugs and risk of dementia overall and by type,^1 4 5^ only a few studies have specifically examined associations between the use of anticholinergic drugs for an overactive bladder and dementia.^38^,^42^ Our finding of an increased risk of dementia with the use of anticholinergic drugs for an overactive bladder is consistent with results from a Canadian population based study that assessed the risk of dementia in patients treated for an overactive bladder with anticholinergic drugs compared with those treated with mirabegron.^38^ Similar findings were reported in a Taiwanese study of 20 246 patients with dementia,^40^ and in a French case-control study.^41^ Although the French study examined the risk of dementia associated with the use of different anticholinergic drugs for an overactive bladder in older adults, the study only assessed the risk of dementia associated with a five year period for the five anticholinergic drugs that have marketing authorisation in France.^41^

The Canadian study assessed an outcome of dementia in patients who were still actively receiving anticholinergic drugs (or mirabegron) for an overactive bladder, or within three months of discontinuing these treatments.^38^ Similar to our study, stronger associations between anticholinergic drugs and dementia were found in men than in women. Unlike the Canadian study, however, we further explored differences in the risk of dementia in men and women by type of anticholinergic drug used to treat an overactive bladder, with findings of similar associations for oxybutynin hydrochloride, but slightly stronger associations in men for solifenacin succinate and tolterodine tartrate. Although the reason for this difference in risk in men and women in unclear, previous research suggests that men and women have different risk profiles for cognitive impairment and progression to dementia.^43^

In a Canadian study by Matta et al, the risk of dementia was examined in 11 392 patients with dementia and in 29 881 matched controls, comparing those who received different antimuscarinic drugs with those who received mirabegron, even up to six months before diagnosis.^42^ Unlike our study, Matta et al did not find a significant increase in the risk of dementia associated with oxybutynin hydrochloride and tolterodine tartrate. These findings are likely attributed to protopathic bias, with the study including treatments for prodromal symptoms of an overactive bladder preceding the diagnosis of dementia.

A population based, drug treatment wide study based on electronic health records from Wales found that dementia was associated with different anticholinergic drug treatments for an overactive bladder, and with mirabegron.^39^ The study, however, had several limitations, such as lack of information on the dose of the prescribed drug or cumulative drug use, and inclusion of prescriptions up to the month before a diagnosis of dementia. These findings could therefore be a result of reverse causation and might explain why associations with dementia diminished in analyses restricted to >5 years and >10 years before diagnosis.^39^

Our finding of an association between anticholinergic drugs used to treat an overactive bladder and a greater increase in the risk of dementia in those aged <80 years at diagnosis is similar to findings from a previous UK study that assessed the risk of dementia associated with anticholinergic drugs in adults aged ≥55 year.^5^ Our study therefore builds on evidence from previous studies and provides new insights on the risk of dementia associated with different anticholinergic drugs used for the treatment of an overactive bladder.

### Study implications for clinicians and policy makers

Our finding that oxybutynin hydrochloride, solifenacin succinate, and tolterodine tartrate were the most frequently prescribed anticholinergic drugs for the treatment of an overactive bladder aligns with findings from a previous study on the use of anticholinergic drugs in older adults in England.^6^ The reason for this prescribing pattern is likely because these medicines have the lowest acquisition cost of all of the anticholinergic drugs used for the treatment of an overactive bladder, and are the first line treatments recommended by national and regional guidelines for the management of an overactive bladder.^9 10^ We found that oxybutynin hydrochloride, solifenacin succinate, and tolterodine tartrate were the anticholinergic drugs most strongly associated with the risk of dementia in older adults. An increased risk of dementia of 25-29% was associated with >1095 total standardised daily doses within a 13 year period for each of these drugs, which is equivalent to three years of daily use of the anticholinergic drug at the standard recommended dose. Our research did not find a significant increase in the risk of dementia with cumulative use of darifenacin, fesoterodine fumarate, flavoxate hydrochloride, propiverine hydrochloride, or trospium chloride. Because of the limited number of patients in some of these subgroups, however, accurately measuring the effects in these groups was restricted and therefore we we cannot conclusively exclude the possibility of a risk of dementia with these drug treatments.

The therapeutic action of anticholinergic drugs in overactive bladder is exerted by blockade of the muscarinic M3 receptors located on the bladder smooth muscle cells.^44^ Muscarinic M1 and M2 receptor subtypes in the brain have an important functional role in cognitive function.^44^ Interactions between anticholinergic drugs and especially M1 receptors in the brain have the potential to cause cognitive impairment, depending on the binding profiles of muscarinic receptors, lipophilicity, and the ability to cross the blood-brain barrier.^45^ Small anticholinergic agents that have a low molecular weight, are lipophilic, and have a neutral charge, such as oxybutynin hydrochloride, can easily cross the blood-brain barrier.^11^ Tolterodine tartrate, fesoterodine fumarate, solifenacin succinate, and darifenacin, similar to oxybutynin hydrochloride, are lipophilic tertiary amines that are partially unpolarised but unlike oxybutynin hydrochloride, their molecules are large.^46^ Trospium chloride is a large, hydrophilic, and positively charged molecule that does not readily cross the blood-brain barrier.

Anticholinergic drugs for the treatment of an overactive bladder also differ in their affinity for the different muscarinic receptor subtypes.^46^ Darifenacin has the highest selectivity for the M3 receptor over the M1 and M2 subtypes, whereas solifenacin succinate has only moderate selectivity for the M3 receptor over the M1 and M2 subtypes. Oxybutynin hydrochloride, fesoterodine fumarate, tolterodine tartrate, trospium chloride, and propiverine hydrochloride have been found to be non-selective for the M3 receptor over the M1 subtype.

Our results might therefore be partly explained by the greater ability of oxybutynin hydrochloride, tolterodine tartrate, and solifenacin succinate to penetrate the blood-brain barrier than other anticholinergic drugs (eg, darifenacin and trospium chloride).^47^ Drugs with greater penetration of the central nervous system are likely to cause more cognitive side effects,^48^ especially in older individuals who have a more permeable blood-brain barrier as well as age related changes in drug metabolism and elimination. In a recent British cohort study, oxybutynin hydrochloride and tolterodine tartrate were found to be associated with more harmful outcomes than other anticholinergic drugs used to treat an overactive bladder when used in patients with dementia.^49^ More careful prescribing of oxybutynin hydrochloride, solifenacin succinate, and tolterodine tartrate by clinicians is therefore needed. Clinical guidelines should recommend alternative treatments that might be associated with a lower risk of dementia, including the use of non-drug interventions, when treating older adults with an overactive bladder. The management of symptoms of an overactive bladder in older adults should include shared decision making between healthcare practitioners and patients that take into account the available treatment options, effectiveness, and possible long term risks and consequences of these treatments.

### Future research

Because mirabegron is rarely prescribed as a first line treatment for older patients with an overactive bladder, and most patients are prescribed anticholinergic drugs before being prescribed mirabegron, an accurate assessment of the independent risk of dementia associated with cumulative use of mirabegron was not possible in this study. Future research should therefore assess the long term effects of cumulative use of mirabegron in older adults who have not previously used anticholinergic drugs to treat an overactive bladder.

### Conclusions

In this study, we found that of the different anticholinergic drugs used to treat an overactive bladder, oxybutynin hydrochloride, solifenacin succinate, and tolterodine tartrate, were most strongly associated with an increased of dementia in older adults. This finding highlights the need for clinicians to take into account the possible long term risks and consequences of the available treatment options for an overactive bladder in older adults, and to consider prescribing alternative treatments that might be associated with a lower risk of dementia.

## Supplementary material

10.1136/bmjmed-2023-000799 online supplemental file 1

## Data Availability

Data may be obtained from a third party and are not publicly available.
