# Supplementary material for: Risk of dementia associated with anticholinergic drugs for overactive bladder in adults aged ≥55 years: nested case-control study
Source: BMJ Med. 2024 Nov 12;3(1):e000799. doi: 10.1136/bmjmed-2023-000799 (PMC11580265; doi:10.1136/bmjmed-2023-000799)
Supplement: online supplemental file 1 [file bmjmed-3-1-s001.pdf]

Supplementary tables and figure

Tables

Table S1. **Defined daily dose (DDD) of overactive bladder drugs**

Overactive bladder drugs	Defined daily dose (DDD)*
Darifenacin hydrobromide	7.5 mg
Fesoterodine fumarate	4 mg
Flavoxate hydrochloride	0.8 g
Oxybutynin hydrochloride	15 mg
Propiverine hydrochloride	30 mg
Solifenacin succinate	5 mg
Tolterodine tartrate	4 mg
Trospium chloride	40 mg
Mirabegron	50 mg

*DDD derived from the WHO collaborating centre for Drugs Statistics Methodology, is the assumed average maintenance dose per day for a drug used for its main indication in adults

Table S2. **Non-OAB anticholinergic drugs and their Anticholinergic Effect on Cognition (AEC) score** [β]

Anticholinergic medication class	AEC score 0	AEC score 1	AEC score 2	AEC score 3	AEC score unknown
Antipsychotics	Amisulpride<br>Haloperidol<br>Lurasidone<br>Risperidone<br>Sulpiride<br>Ziprasidone	Aripiprazole<br>Fluphenazine<br>Iloperidone<br>Perphenazine<br>Sertindole<br>Flupenthixol<br>Paliperidone<br>Zuclopentixol	Levomepromazine<br>Olanzapine<br>Pimozide<br>Prochlorperazine<br>Promazine<br>Quetiapine<br>Trifluoperazine<br>Zotepine	Chlorpromazine<br>Clozapine	
Antidepressants	Reboxetine<br>Trazodone<br>Venlafaxine<br>Vortioxetine<br>Bupropion<br>Duloxetine<br>Fluvoxamine<br>Moclobemide	Citalopram<br>Fluoxetine<br>Lithium<br>Mirtazapine<br>Sertraline<br>Buspirone<br>Phenelzine<br>Isocarboxazid	Desipramine<br>Paroxetine	Amitriptyline<br>Clomipramine<br>Dothiepin<br>Doxepin<br>Imipramine<br>Lofepramine<br>Nortriptyline<br>Trimipramine	
Gastrointestinal drugs (antiemetics and antispasmodics)	Alverine<br>Cimetidine<br>Docusate sodium<br>Gaviscon<br>Granisetron<br>Lactulose<br>Loperamide<br>Macrogol<br>Mebeverine<br>Mesalazine<br>Metoclopramide<br>Omeprazole<br>Ondansetron<br>Peppermint oil<br>Pantoprazole<br>Rabeprazole<br>Ranitidine<br>Bisacodyl<br>Senna<br>Sulphasalazine	Hyoscine<br>Pirenzepine<br>Domperidone	Dicycloverine<br>Propantheline<br>Dimenhydrinate	Hyoscine<br>Hydrobromide	Lansoprazole
Anti-epileptics	Gabapentin<br>Lamotrigine<br>Valproate	Carbamazepine			Levetiracetam<br>Oxcarbazepine<br>Pregabalin<br>Phenytoin
Other anticholinergic drugs	Alendronic acid<br>Alprazolam<br>Amlodipine<br>Amoxicillin<br>Aspirin<br>Atenolol<br>Atorvastatin<br>Cephalexin<br>Cetirizine<br>Chlordiazepoxide<br>Ciprofloxacin<br>Clopidogrel	Amiodarone<br>Bromocriptine<br>Diazepam<br>Fentanyl<br>Hydroxyzine<br>Lithium<br>Prednisolone<br>Quinidine<br>Temazepam	Amantadine<br>Chlorphenamine<br>Diphenhydramine<br>Disopyramide<br>Pethidine	Alimenazine<br>Atropine<br>Benztropine<br>Clemastine<br>Cyproheptadine<br>Orphenadrine<br>Procyclidine<br>Promethazine<br>Trihexyphenidryl (benzhexol)	Alendronic acid<br>Allopurinol<br>Anastrozole<br>Apixaban<br>Baclofen<br>Bisoprolol<br>Bumetanide<br>Captopril<br>Carbimazole<br>Carvedilol<br>Chlortalidone<br>Clarithromycin

	Diclofenac<br>Diltiazem<br>Enalapril<br>Entacapone<br>Fexofenadine<br>Furosemide<br>Gliclazide<br>Ibuprofen<br>Ketorolac<br>Levadopa<br>Lisinopril<br>Loratadine<br>Lorazepam<br>Losartan<br>Lovastatin<br>Meloxicam<br>Metoprolol<br>Morphine<br>Naproxen<br>Paracetamol<br>Pravastatin<br>Propranolol<br>Rosiglitazone<br>Simvastatin<br>Theophylline<br>Thyroxine<br>Tramadol<br>Trimethoprim<br>Warfarin<br>Zolpidem				Codeine<br>Colchicine<br>Dabigatran<br>Dexamethasone<br>Dextropropoxyphene<br>Digoxin<br>Erythromycin<br>Hydrocodone<br>Irbesartan<br>Metformin<br>Methocarbamal<br>Methotrexate<br>Nitrofurantoin<br>Oxycodone<br>Ramipril<br>Rivaroxaban<br>Rovustatin<br>Spironolactone<br>Tamoxifen<br>Topiramate<br>Tizanidine<br>Verapamil<br>Zopiclone

[β] Anticholinergic Effect on Cognition (AEC) score derived from Bishara et al. (22)

Table S3. **Baseline characteristics of primary care patients in CPRD prescribed bladder anticholinergic drugs and Mirabegron**

	Bladder anticholinergic drugs n(%)								
Patient characteristics	Darifenacin (n=332)	Fesoterodine (n=2,646)	Flavoxate (n=2,599)	Oxybutynin (n=40,531)	Propiverine (n=2,193)	Solifenacin (n=23,836)	Tolterodine (n=35,240)	Trospium (n=7,320)	Mirabegron (n=1,826)
**Age (years) at first prescription of drug, mean(SD)**									
Overall	77.4 (6.6)	76.2 (7.3)	71.6 (8.7)	72.3 (8.9)	72.9 (8.0)	76.1 (7.4)	73.7 (8.1)	74.4 (7.8)	77.9 (6.8)
Patients with dementia (cases)	78.1 (6.4)	76.4 (7.3)	72.1 (9.0)	72.6 (9.0)	72.6 (8.0)	75.8 (7.7)	73.6 (8.3)	74.0 (8.1)	77.1 (7.1)
Patients without dementia (controls)	77.3 (6.7)	75.4 (7.5)	71.5 (8.7)	72.3 (8.8)	73.0 (8.0)	76.2 (7.3)	73.7 (8.0)	74.5 (7.7)	78.2 (6.7)
p-value[#]	0.3999	0.0059	0.1397	0.0290	0.2847	0.0020	0.1090	0.0290	0.0036
**Sex**									
Female	256 (77.1)	1,912 (72.3)	1,720 (66.2)	28,133 (69.4)	1,697 (77.4)	17,168 (72.0)	25,145 (71.4)	5,367 (73.3)	1,226 (67.1)
Male	76 (22.9)	734 (27.7)	879 (33.8)	12,398 (30.6)	496 (22.6)	6,668 (28.0)	10,095 (28.7)	1,953 (26.7)	600 (32.9)
p-value[~]	<0.005	<0.005	<0.005	<0.005	<0.005	<0.005	<0.005	<0.005	<0.005
**Level of Practice deprivation (IMD) (N=447,483)**									
	n=125 (100)	n=584 (100)	n=1,411 (100)	n=18,423	n=739	n=6,727	n=14,048	n=2,615	n=28
1 (least deprived)	31 (24.8)	73 (12.5)	241 (17.1)	2,701 (14.7)	99 (13.4)	1,069 (15.9)	2,459 (17.5)	355 (13.6)	28 (10.7)
2	10 (8.0)	86 (14.7)	281 (19.9)	3,676 (20.0)	150 (20.3)	1,485 (22.1)	3,071 (21.9)	584 (22.3)	65 (24.7)
3	18 (14.4)	100 (17.1)	230 (16.3)	3,302 (17.9)	138 (18.7)	1,204 (17.9)	2,377 (16.9)	425 (16.3)	41 (15.6)
4	31 (24.8)	144 (24.7)	289 (20.5)	4,195 (22.8)	169 (22.9)	1,319 (19.6)	2,889 (20.6)	443 (16.9)	43 (16.4)
5 (most deprived)	35 (28.0)	181 (31.0)	370 (26.2)	4,549 (24.7)	183 (24.8)	1,650 (24.5)	3,252 (23.2)	808 (30.9)	86 (32.7)
p-value (for trend)[+]	0.001	<0.001	0.010	<0.001	0.091	<0.001	<0.001	<0.001	<0.001

[#]p-value for test of significance when comparing difference in age of first prescription of the OAB drug types in cases and controls

[~]p-value for test of significance when comparing difference in prescribing of the OAB drug types in males and females

[+]p-value for test of significance in difference in trends of prescribing of the OAB drug types by IMD groups

Table S4a. **Crude and adjusted odds ratios for dementia¥ by total cumulative use of different bladder anticholinergic drugs and mirabegron, in subgroup of patients aged 80 years and over (n=645,201)**

Anticholinergic medication	Cumulative exposure to anticholinergic (TSDD)	Patients with dementia (Cases, n =115,369)	Patients without dementia (Controls, n = 529,832)	Unadjusted odds ratio (95% CI)	Adjusted odds ratio** (95% CI)
Darifenacin hydrobromide	None	115,319 (99.96)	529,643 (99.96)	1.00 [Reference]	1.00 [Reference]
	1-90	26 (0.02)	92 (0.02)	1.32 (0.85-2.05)	1.16 (0.74-1.82)
	91-365	13 (0.01)	51 (0.01)	1.15 (0.62-2.12)	1.08 (0.58-2.02)
	366-1095	7 (0.01)	32 (0.01)	0.88 (0.38-2.04)	0.88 (0.38-2.05
	>1095	4 (0.00)	14 (0.00)	1.43 (0.47-4.34)	1.58 (0.51-4.87)
Fesoterodine fumarate	None	115,045 (99.72)	528,385 (99.73)	1.00 [Reference]	1.00 [Reference]
	1-90	130 (0.11)	673 (0.13)	0.86 (0.71-1.04)	0.82 (0.67-0.99)
	91-365	90 (0.08)	363 (0.07)	1.16 (0.92-1.46)	1.06 (0.84-1.35)
	366-1095	64 (0.06)	242 (0.05)	1.17 (0.88-1.54)	1.07 (0.81-1.42)
	>1095	40 (0.03)	169 (0.03)	1.05 (0.74-1.48)	0.93 (0.65-1.32)
Flavoxate hydrochloride	None	114,996 (99.68)	528,220 (99.70)	1.00 [Reference]	1.00 [Reference]
	1-90	290 (0.25)	1,277 (0.24)	1.02 (0.90-1.17)	0.95 (0.83-1.08)
	91-365	48 (0.04)	194 (0.04)	1.13 (0.82-1.56)	1.04 (0.75-1.44)
	366-1095	24 (0.02)	108 (0.02)	1.01 (0.65-1.59)	0.98 (0.62-1.55)
	>1095	11 (0.01)	33 (0.01)	1.57 (0.79-3.11)	1.40 (0.70-2.82)
Oxybutynin hydrochloride	None	109,724 (95.11)	505,641 (95.43)	1.00 [Reference]	1.00 [Reference]
	1-90	3,775 (3.27)	16,728 (3.16)	1.03 (1.00-1.07)	1.03 (0.99-1.07)
	91-365	979 (0.85)	4,045 (0.76)	1.09 (1.02-1.17)	1.09 (1.01-1.17)
	366-1095	618 (0.54)	2,292 (0.43)	1.23 (1.12-1.34)	1.22 (1.12-1.34)
	>1095	273 (0.24)	1,126 (0.21)	1.11 (0.97-1.27)	1.09 (0.95-1.25)
Propiverine hydrochloride	None	115,058 (99.73)	528,588 (99.77)	1.00 [Reference]	1.00 [Reference]
	1-90	164 (0.14)	704 (0.13)	1.07 (0.90-1.27)	1.00 (0.84-1.19)
	91-365	53 (0.05)	209 (0.04)	1.13 (0.84-1.54)	1.02 (0.75-1.39)
	366-1095	50 (0.04)	180 (0.03)	1.29 (0.94-1.77)	1.16 (0.84-1.59)
	>1095	44 (0.04)	151 (0.03)	1.34 (0.96-1.88)	1.25 (0.89-1.77)
Solifenacin succinate	None	112,168 (97.23)	516,238 (97.43)	1.00 [Reference]	1.00 [Reference]
	1-90	1,238 (1.07)	5,747 (1.08)	0.98 (0.92-1.04)	0.99 (0.93-1.05)

	91-365	721 (0.62)	3,156 (0.60)	1.04 (0.96-1.13)	1.03 (0.95-1.12)
	366-1095	644 (0.56)	2,531 (0.48)	1.15 (1.05-1.25)	1.14 (1.04-1.25)
	>1095	598 (0.52)	2,160 (0.41)	1.26 (1.15-1.38)	1.24 (1.12-1.36)
Tolterodine tartrate	None	110,499 (95.87)	509,183 (96.10)	1.00 [Reference]	1.00 [Reference]
	1-90	2,275 (1.97)	10,228 (1.93)	1.01 (0.96-1.06)	1.02 (0.97-1.07)
	91-365	1,068 (0.93)	4,401 (0.83)	1.11 (1.04-1.19)	1.11 (1.03-1.19)
	366-1095	789 (0.68)	3,004 (0.57)	1.20 (1.11-1.30)	1.21 (1.11-1.31)
	>1095	738 (0.64)	3,016 (0.57)	1.11 (1.03-1.21)	1.13 (1.04-1.23)
Trospium chloride	None	114,335 (99.20)	525,609 (99.20)	1.00 [Reference]	1.00 [Reference]
	1-90	547 (0.47)	2,400 (0.45)	1.05 (0.96-1.16)	0.98 (0.89-1.08)
	91-365	241 (0.21)	913 (0.17)	1.21 (1.05-1.40)	1.13 (0.98-1.32)
	366-1095	165 (0.14)	556 (0.10)	1.36 (1.14-1.62)	1.25 (1.04-1.50)
	>1095	81 (0.07)	354 (0.07)	1.03 (0.81-1.32)	0.99 (0.77-1.27)
Mirabegron	None	115,096 (99.76)	528,838 (99.81)	1.00 [Reference]	1.00 [Reference]
	1-90	94 (0.08)	459 (0.09)	0.91 (0.73-1.14)	0.82 (0.66-1.04)
	91-365	95 (0.08)	309 (0.06)	1.36 (1.08-1.71)	1.27 (1.00-1.62)
	366-1095	76 (0.07)	191 (0.04)	1.82 (1.39-2.38)	1.66 (1.26-2.19)
	>1095	8 (0.01)	35 (0.01)	1.02 (0.47-2.22)	0.96 (0.44-2.10)

[¥]Dementia outcome includes all dementia subtypes.

**Multivariable model adjusted for ethnicity, patient level of deprivation, smoking status, alcohol consumption, body mass index, records of anxiety, asthma, atrial fibrillation, bipolar disorder, chronic kidney disease, chronic obstructive pulmonary disease (COPD), coronary heart disease, depression, down syndrome, head injury, heart failure, hyperlipidaemia, hypertension, learning disability, schizophrenia, stroke, subarachnoid haemorrhage, transient ischaemic attack (TIA), type 1 diabetes, type 2 diabetes, mean anticholinergic effect of cognition (AEC) score of other prescribed non-bladder anticholinergic medication, prescription of other commonly prescribed bladder anticholinergic medication (oxybutynin, solifenacin, tolterodine); with case-control matching by age, sex and general practice.

Table S4b. **Crude and adjusted odds ratios for dementia¥ by total cumulative use of different bladder anticholinergic drugs and mirabegron, in subgroup of patients < 80 years (n=329,926)**

Anticholinergic medication	Cumulative exposure to anticholinergic (TSDD)	Patients with dementia (Cases, n =55,373)	Patients without dementia (Controls, n = 274,553)	Unadjusted odds ratio (95% CI)	Adjusted odds ratio** (95% CI)
Darifenacin hydrobromide	None	55,357 (99.97)	274,476 (99.97)	1.00 [Reference]	1.00 [Reference]
	1-90	10 (0.02)	35 (0.01)	1.40 (0.69-2.83)	0.93 (0.44-1.94)
	91-365	2 (0.00)	19 (0.01)	0.53 (0.12-2.26)	0.38 (0.09-1.67)
	366-1095	3 (0.01)	19 (0.01)	0.75 (0.22-2.57)	0.60 (0.17-2.12)
	>1095	1 (0.00)	4 (0.00)	1.27 (1.14-11.36)	0.41 (0.04-4.07)
Fesoterodine fumarate	None	55,169 (99.63)	273,882 (99.76)	1.00 [Reference]	1.00 [Reference]
	1-90	90 (0.16)	324 (0.12)	1.37 (1.09-1.74)	1.03 (0.80-1.32)
	91-365	48 (0.09)	167 (0.06)	1.43 (1.04-1.98)	1.12 (0.80-1.57)
	366-1095	35 (0.06)	107 (0.04)	1.64 (1.12-2.40)	1.16 (0.78-1.74)
	>1095	31 (0.06)	73 (0.03)	2.15 (1.41-3.28)	1.68 (1.07-2.63)
Flavoxate hydrochloride	None	55,253 (99.78)	274,059 (99.82)	1.00 [Reference]	1.00 [Reference]
	1-90	89 (0.16)	398 (0.14)	1.11 (0.88-1.40)	0.84 (0.66-1.07)
	91-365	14 (0.03)	62 (0.02)	1.13 (0.63-2.01)	0.84 (0.46-1.53)
	366-1095	8 (0.01)	22 (0.01)	1.82 (0.81-4.09)	1.58 (0.68-3.70)
	>1095	9 (0.02)	12 (0.00)	3.71 (1.56-8.81)	2.72 (1.07-6.97)
Oxybutynin hydrochloride	None	53,066 (95.83)	266,165 (96.94)	1.00 [Reference]	1.00 [Reference]
	1-90	1,435 (2.59)	5,938 (2.16)	1.22 (1.15-1.29)	1.07 (1.01-1.14)
	91-365	414 (0.75)	1,316 (0.48)	1.59 (1.42-1.77)	1.32 (1.17-1.49)
	366-1095	283 (0.51)	739 (0.27)	1.93 (1.68-2.21)	1.60 (1.38-1.85)
	>1095	175 (0.32)	395 (0.14)	2.23 (1.87-2.67)	1.86 (1.53-2.25)
Propiverine hydrochloride	None	55,216 (99.72)	274,072 (99.82)	1.00 [Reference]	1.00 [Reference]
	1-90	77 (0.14)	270 (0.10)	1.42 (1.10-1.83)	1.07 (0.82-1.40)
	91-365	39 (0.07)	96 (0.03)	2.02 (1.39-2.93)	1.47 (0.99-2.19)
	366-1095	26 (0.05)	66 (0.02)	1.96 (1.24-3.08)	1.16 (0.71-1.89)
	>1095	15 (0.03)	49 (0.02)	1.54 (0.86-2.74)	1.08 (0.58-1.99)
Solifenacin succinate	None	53,796 (97.15)	269,089 (98.01)	1.00 [Reference]	1.00 [Reference]
	1-90	551 (1.00)	2,163 (0.79)	1.28 (1.17-1.41)	1.12 (1.01-1.24)

	91-365	388 (0.70)	1,288 (0.47)	1.52 (1.36-1.70)	1.32 (1.17-1.49)
	366-1095	308 (0.56)	1,027 (0.37)	1.51 (1.33-1.72)	1.23 (1.07-1.41)
	>1095	330 (0.60)	986 (0.36)	1.69 (1.49-1.92)	1.37 (1.19-1.56)
Tolterodine tartrate	None	53,183 (96.05)	267,022 (97.26)	1.00 [Reference]	1.00 [Reference]
	1-90	961 (1.74)	3,823 (1.39)	1.26 (1.18-1.36)	1.14 (1.06-1.23)
	91-365	497 (0.90)	1,665 (0.61)	1.51 (1.36-1.67)	1.26 (1.13-1.40)
	366-1095	364 (0.66)	1,050 (0.38)	1.74 (1.54-1.96)	1.49 (1.31-1.70)
	>1095	368 (0.66)	993 (0.36)	1.87 (1.66-2.11)	1.60 (1.41-1.82)
Trospium chloride	None	54,889 (99.13)	272,974 (99.42)	1.00 [Reference]	1.00 [Reference]
	1-90	234 (0.42)	892 (0.32)	1.30 (1.13-1.51)	1.02 (0.88-1.19)
	91-365	115 (0.21)	363 (0.13)	1.57 (1.27-1.93)	1.09 (0.87-1.36)
	366-1095	79 (0.14)	179 (0.07)	2.20 (1.69-2.87)	1.53 (1.16-2.03)
	>1095	56 (0.10)	145 (0.05)	1.89 (1.39-2.58)	1.37 (0.99-1.90)
Mirabegron	None	55,230 (99.74)	274,137 (99.85)	1.00 [Reference]	1.00 [Reference]
	1-90	58 (0.10)	188 (0.07)	1.54 (1.14-2.06)	1.14 (0.83-1.56)
	91-365	40 (0.07)	111 (0.04)	1.76 (1.23-2.53)	1.26 (0.86-1.85)
	366-1095	40 (0.07)	97 (0.04)	2.05 (1.42-2.96)	1.52 (1.04-2.24)
	>1095	5 (0.01)	20 (0.01)	1.26 (0.47-3.36)	0.71 (0.25-2.02)

[¥]Dementia outcome includes all dementia subtypes.

**Multivariable model adjusted for ethnicity, patient level of deprivation, smoking status, alcohol consumption, body mass index, records of anxiety, asthma, atrial fibrillation, bipolar disorder, chronic kidney disease, chronic obstructive pulmonary disease (COPD), coronary heart disease, depression, down syndrome, head injury, heart failure, hyperlipidaemia, hypertension, learning disability, schizophrenia, stroke, subarachnoid haemorrhage, transient ischaemic attack (TIA), type 1 diabetes, type 2 diabetes, mean anticholinergic effect of cognition (AEC) score of other prescribed non-bladder anticholinergic medication, prescription of other commonly prescribed bladder anticholinergic medication (oxybutynin, solifenacin, tolterodine); with case-control matching by age, sex and general practice.

Table S5. **Adjusted odds ratios for dementia associated with different bladder anticholinergic drugs and mirabegron, in males and females**

Anticholinergic medication	Cumulative exposure to anticholinergic (TSDD)	Adjusted odds ratio** (95% CI) in males (n=363,213)	Adjusted odds ratio** (95% CI) in females (n=611,914)
		Cases = 63,865<br>Controls = 299,348	Cases = 106,877<br>Controls = 505,037
All bladder anticholinergic medications		1.22 (1.18-1.26)	1.16 (1.13-1.19)
Darifenacin hydrobromide	None	1.00	1.00
	1-90	1.41 (0.69-2.89)	0.99 (0.63-1.56)
	91-365	0.33 (0.04-2.61)	0.99 (0.55-1.81)
	366-1095	2.29 (0.80-6.57)	0.40 (0.14-1.15)
	>1095	1.36 (0.10-17.9)	1.25 (0.41-3.80)
Fesoterodine fumarate	None	1.00	1.00
	1-90	1.07 (0.82-1.41)	0.84 (0.70-1.01)
	91-365	1.36 (0.98-1.90)	0.96 (0.76-1.22)
	366-1095	1.11 (0.72-1.72)	1.10 (0.84-1.45)
	>1095	1.42 (0.87-2.32)	1.06 (0.76-1.48)
Flavoxate hydrochloride	None	1.00	1.00
	1-90	0.87 (0.71-1.06)	0.93 (0.80-1.07)
	91-365	1.14 (0.72-1.82)	0.87 (0.61-1.25)
	366-1095	1.36 (0.75-2.46)	0.88 (0.51-1.53)
	>1095	1.94 (0.75-5.03)	1.62 (0.83-3.17)
Oxybutynin hydrochloride	None	1.00	1.00
	1-90	1.03 (0.97-1.09)	1.05 (1.01-1.09)
	91-365	1.14 (1.02-1.27)	1.14 (1.06-1.23)
	366-1095	1.24 (1.08-1.42)	1.35 (1.23-1.48)
	>1095	1.28 (1.06-1.55)	1.29 (1.13-1.47)
Propiverine hydrochloride	None	1.00	1.00
	1-90	1.12 (0.84-1.48)	0.99 (0.84-1.18)
	91-365	1.27 (0.79-2.05)	1.14 (0.87-1.51)
	366-1095	1.21 (0.63-2.32)	1.19 (0.89-1.59)
	>1095	1.24 (0.63-2.42)	1.17 (0.83-1.64)
Solifenacin succinate	None	1.00	1.00
	1-90	1.12 (1.01-1.24)	0.99 (0.93-1.05)
	91-365	1.27 (1.12-1.43)	1.05 (0.97-1.15)
	366-1095	1.32 (1.16-1.51)	1.11 (1.02-1.22)
	>1095	1.49 (1.30-1.72)	1.20 (1.10-1.32)
Tolterodine tartrate	None	1.00	1.00
	1-90	1.09 (1.01-1.17)	1.03 (0.98-1.08)
	91-365	1.33 (1.20-1.47)	1.08 (1.00-1.16)
	366-1095	1.45 (1.28-1.64)	1.21 (1.12-1.32)
	>1095	1.23 (1.08-1.40)	1.26 (1.16-1.37)
Trospium chloride	None	1.00	1.00
	1-90	1.13 (0.97-1.32)	0.94 (0.86-1.04)
	91-365	1.63 (1.33-2.01)	0.93 (0.80-1.09)
	366-1095	1.26 (0.94-1.69)	1.33 (1.11-1.59)
	>1095	1.49 (1.03-2.15)	1.002 (0.81-1.28)
Mirabegron	None	1.00	1.00
	1-90	1.28 (0.94-1.76)	0.78 (0.62-0.99)

	91-365	1.53 (1.12-2.09)	1.10 (0.84-1.44)
	366-1095	1.46 (1.00-2.13)	1.72 (1.30-2.27)
	>1095	0.55 (0.16-1.87)	1.13 (0.54-2.34)

**Multivariable model adjusted for ethnicity, patient level of deprivation, smoking status, alcohol consumption, body mass index, records of anxiety, asthma, atrial fibrillation, bipolar disorder, chronic kidney disease, chronic obstructive pulmonary disease (COPD), coronary heart disease, depression, down syndrome, head injury, heart failure, hyperlipidaemia, hypertension, learning disability, schizophrenia, stroke, subarachnoid haemorrhage, transient ischaemic attack (TIA), type 1 diabetes, type 2 diabetes, mean anticholinergic effect of cognition (AEC) score of other prescribed non-bladder anticholinergic medication, prescription of other commonly prescribed bladder anticholinergic medication (oxybutynin, solifenacin, tolterodine); with case-control matching by age, sex and general practice.

Table S6.

Crude and adjusted odds ratios for dementia[¥] by total cumulative use of mirabegron in patients with no bladder anticholinergic drug exposure, 3-16 years before dementia[¥] diagnosis

Anticholinergic medication	Cumulative exposure to anticholinergic (TSDD)	Cases (n =170,376)	Controls (n = 803,158)	Unadjusted odds ratio (95% CI)	Adjusted odds ratio** (95% CI)
Mirabegron	None	170,317 (99.97)	802,965 (99.98)	1.00 [Reference]	1.00 [Reference]
	1-90	22 (0.01)	98 (0.01)	1.06 (0.67-1.69)	1.09 (0.68-1.76)
	91-365	22 (0.01)	54 (0.01)	1.93 (1.17-3.18)	1.99 (1.19-3.31)
	366->1095***	15 (0.01)	41 (0.01)	1.73 (0.95-3.13)	1.81 (0.98-3.33)

[¥]Dementia outcome includes all dementia subtypes.

**Multivariable model adjusted for ethnicity, patient level of deprivation, smoking status, alcohol consumption, body mass index, records of anxiety, asthma, atrial fibrillation, bipolar disorder, chronic kidney disease, chronic obstructive pulmonary disease (COPD), coronary heart disease, depression, down syndrome, head injury, heart failure, hyperlipidaemia, hypertension, learning disability, schizophrenia, stroke, subarachnoid haemorrhage, transient ischaemic attack (TIA), type 1 diabetes, type 2 diabetes, mean anticholinergic effect of cognition (AEC) score of other prescribed non-bladder anticholinergic medication; with case-control matching by age, sex and general practice.

***last row for TSDD >1095 has been suppressed and merged with TSDD category 366-1095 due to small cell count with fewer than 5 observations

Supplementary figures

Figure S1.

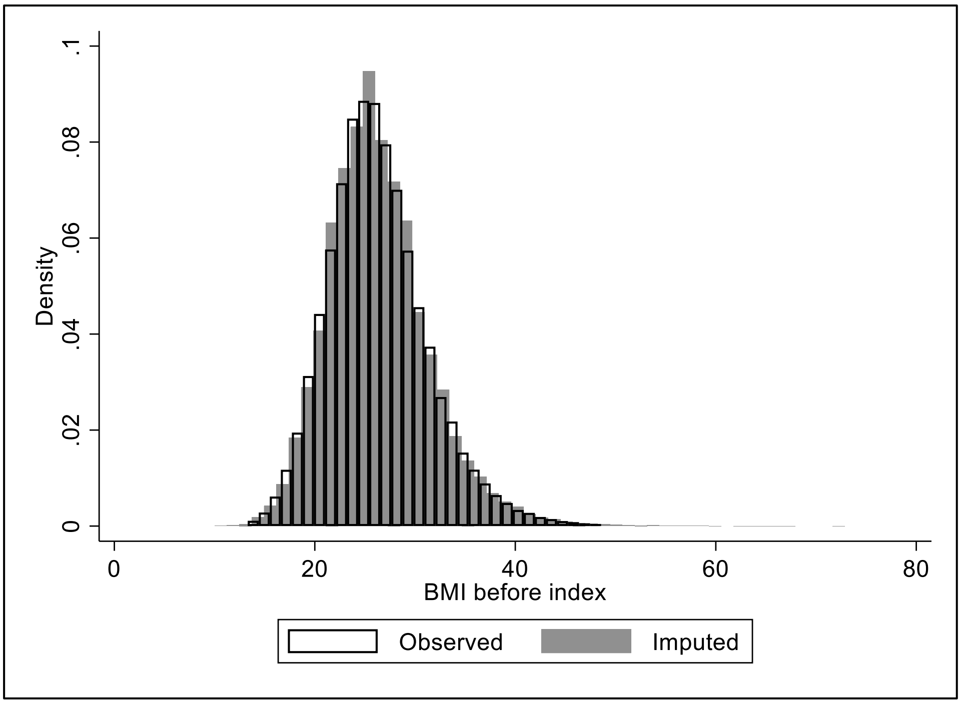

Figure S1. Kernel density plot showing the distribution of observed BMI records superimposed on the plot of BMI records after data imputation.
