# Supplementary material for: Risk of dementia associated with anticholinergic drugs for overactive bladder in adults aged ≥55 years: nested case-control study
Source: BMJ Med. 2024 Nov 12;3(1):e000799. doi: 10.1136/bmjmed-2023-000799 (PMC11580265; doi:10.1136/bmjmed-2023-000799)
Supplement: Supplementary file 2 [file bmjmed-2023-000799.reviewer_comments.pdf]

PEER REVIEW HISTORY

BMJ Medicine publishes all reviews undertaken for accepted manuscripts. Reviewers are asked to complete a checklist review form and are provided with free text boxes to elaborate on their assessment. These free text comments are reproduced below.

ARTICLE DETAILS

**TITLE (PROVISIONAL)**	Risk of dementia associated with anticholinergic drugs for overactive bladder: a nested case-control study of adults aged 55 years and over
**AUTHORS**	Iyen, Barbara; Bell, Brian; Coupland, Carol; Ashcroft, Darren; Bishara, Delia; Orrell, Martin; Dening, Tom; Avery, Anthony

VERSION 1 - REVIEW

**REVIEWER NAME**	Nishtala, Prasad S.
**REVIEWER AFFILIATION**	University of Bath, Life Sciences
**REVIEWER CONFLICT OF INTEREST**	None
**DATE REVIEW RETURNED**	22-Nov-2023

**GENERAL COMMENTS**	The authors have investigated the differential risks for dementia posed by bladder anticholinergics in a large representative population of adults 55 years and above extracted from the CPRD database. I have a few comments for their consideration.<br><br>Exposure Classification: I am unfamiliar with the literature on using Total Standardised Daily Dose (TSDD) for classifying anticholinergic exposure. It's essential to qualify this choice with appropriate references that link TSDD thresholds to clinical outcome risks. This is particularly important given the established relevance of the Drug Burden Index in pharmacological studies. (Nishtala PS, Allore H, Han L, Jamieson HA, Hilmer SN, Chyou TY. Impact of Anticholinergic Burden on Cognitive Performance: A Cohort Study of Community-Dwelling Older Adults. J Am Med Dir Assoc. 2020 Sep;21(9):1357-1358.e3. doi: 10.1016/j.jamda.2020.03.027. Epub 2020 May 10. PMID: 32402780; PMCID: PMC7971451). Clarifying why TSDD is a good measure of anticholinergic exposure would improve the paper's validity.<br>Strengths of the Study: The methodology adopted by the authors to mitigate protopathic bias, notably excluding exposures three years before the index date, is commendable. This approach significantly strengthens the study's findings. The differential risk for delirium with anticholinergics(Nishtala PS, Chyou TY. Risk of delirium associated with antimuscarinics in older adults: A case-time-control study. Pharmacoepidemiol Drug Saf. 2022 Aug;31(8):883-891. doi: 10.1002/pds.5480. Epub 2022 May 25. PMID: 35587029; PMCID: PMC9545361.)has been studied previously. I agree with the novelty of this research in comparing the differential risk of dementia associated with various anticholinergics. Another noteworthy aspect is the use of mirabegron as a non-anticholinergic comparator for overactive bladder (OAB) drugs, enhancing the study's robustness.<br>Addressing Confounders: Can the authors clarify if they accounted for the use of over-the-counter (OTC) antihistamines, which could potentially misclassify exposure? It should be noted as a limitation if

	this hasn't been considered.<br>Limitations: The manuscript would benefit from a more thorough discussion of the baseline differences between cases and non-cases, particularly in terms of BMI, alcohol consumption, smoking status, and depression. It's unclear whether covariate adjustment using propensity scores was considered to address these differences. Moreover, a deeper exploration of muscarinic receptor selectivity, beyond permeability to the blood-brain barrier (BBB), could provide a more nuanced understanding of the central anticholinergic adverse effects of antimuscarinics. The manuscript presents a somewhat oversimplified view of bladder anticholinergics' biological mechanisms and differential risks.<br>Overall Assessment: This is a well-conducted, large-scale pharmacoepidemiological study that contributes valuable insights to the existing evidence on the differential risks posed by anticholinergics concerning dementia.

**REVIEWER NAME**	Mattishent, Katharina
**REVIEWER AFFILIATION**	University of East Anglia
**REVIEWER CONFLICT OF INTEREST**	None
**DATE REVIEW RETURNED**	30-Nov-2023

**GENERAL COMMENTS**	Thank you for the opportunity to review this manuscript.<br><br>The authors have conducted a very interesting study with mostly sound methodology.<br><br>1. Could the authors discuss the validity of the Mirabegron data in more depth, please. There appears to be unmeasured confounding with resulted in the significant risk in dementia. Mirabegron is used 2nd or 3rd line after a patient will have been taking for example Oxybutinin or Solifenacin for some time. Do the authors think the results are due to protopathic bias or confounding by indication?<br><br>2. The drugs with insignificant findings happen to be the ones with very few numbers of patient taking them, hence also wide Confidence Intervals. Arguably, it is tricky to definitely rule out risk of dementia. These drugs may not be safer alternatives both from the risk of dementia aspect and other anticholinergic adverse effects.<br><br>Thank you.

**REVIEWER NAME**	Trenaman, Shanna C.
**REVIEWER AFFILIATION**	Dalhousie University, College of Pharmacy
**REVIEWER CONFLICT OF INTEREST**	None
**DATE REVIEW RETURNED**	14-Dec-2023

**GENERAL COMMENTS**	I would like to thank the authors for this large, rigorous, observational study of the relationship between bladder anticholinergics and risk of dementia. I read the paper with great interest and have followed this line of inquiry for some time so I am familiar with the literature base. Given the pre-existing studies in this area I was surprised that the authors did not stratify the population

	by sex or by age of exposure > or < 80 years given the known differences in dementia risk in these populations. Is there a reason that this was not added, or commented on? I also think that the context of the discussion was too short and I would like to hear more about the gap that this particular analysis fills in relation to the previous studies on this topic.

**REVIEWER NAME**	Juurlink, David
**REVIEWER AFFILIATION**	Sunnybrook Research Institute
**REVIEWER CONFLICT OF INTEREST**	None
**DATE REVIEW RETURNED**	16-Dec-2023

**GENERAL COMMENTS**	This review was done jointly by Carolyn Tan MD and David Juurlink MD<br><br>This nested case-control study explores the association between drugs for OAB and incident dementia.<br><br>GENERAL COMMENTS<br><br>- This is an important topic and the manuscript is generally well written.<br><br>- Reading the results, the main question is "Are these associations likely to be causal?" In favour of cause-effect are plausibility and temporality. But the lack of a compelling gradient in almost every analysis (adjusted ORs in Table 4) and the finding of associations where they are not expected (eg - for trospium, which should not cross the BBB, and for mirabegron, the notional comparator) seriously undermine the case for causation, especially in light of the modest effect sizes generated. There is no obvious remedy to this.<br><br>- The inclusion of younger cases (55-65) may adversely influence the signal:noise in this analysis because such patients are more likely to have a genetic cause of dementia (APP, PSEN1/2) rather than a drug-related component.<br><br>- It's not clear whether controls were permitted to become cases at later points in time, as should be the case<br><br>- We understand the reason for not including the 3 years prior to the index in the exposure assessment, but there might be value in including it in a sensitivity analysis<br><br>SPECIFIC COMMENTS<br><br>ABSTRACT<br><br>Page 3<br>Line 28 – should be “TSDD” instead of “TSSD”<br>Line 48 – had to re-read as it wasn’t clear what the 2nd AOR was referring to until the end of the sentence. Please reword.<br><br>Page 4<br>Line 5 – “trend of association” – it is not clear what this means (same wording used page 17 line 26) and I don’t think this conclusion can be drawn based on the available results

Line 41 - median/IQR preferable to mean/SD

INTRODUCTION

Page 5
Line 13 – sounds like the “older population” refers to those > 20 years of age. Reword to: “Despite this, use of these drugs nearly doubled among older adults in England over the last 20 years (6).”
Line 18 – consider removing “debilitating” and “(in the absence of infection)”
Line 40 – anticholinergic drugs for OAB also differ in their affinity for M1 receptors in the brain. Please comment on this.
Line 45 – potential contribution of oxybutynin to neurofibrillary tangles and amyloid plaques is not mentioned in reference 11. Where is this information from? Per Welk 2021 (see references), data on association between anticholinergic drugs and presence of plaques and tangles is mixed.
Line 58 – what does “more reassuring” mean? Was there no association with cognitive decline? If so, please state this explicitly.
Please include mechanism of mirabegron and rationale for why it has been included in the study

METHODS

Page 7
Line 29 – does this mean that controls could have a diagnosis of mild cognitive impairment or not otherwise specified cognitive decline?
Line 42 – missing “as” before “this enabled exposure”

Page 8
Line 24 – should be “TSDD” instead of “TSSD”
In keeping with RECORD-PE item 7.1.g, please specify how participants with exposure to multiple OAB drugs were handled? From Table 4 it appears that they were included for each individual drug, meaning that participants may be counted > 1 time(s)

Page 9
Please separate out variables known to increase risk of dementia and indications for other anticholinergic drug use
Line 15 – “and” is missing before “type 2 diabetes”
Line 26 – “We also accounted for patients’ use of commonly prescribed overactive bladder anticholinergic drugs” – why is this included under the covariates section?
Line 38 – “…and academics with experience of research of anticholinergic drugs research” – typo

Page 10
Include use of Pearson’s chi-squared test for baseline characteristics
Line 27 – “…assumptions were made that individuals with missing or unrecorded clinical variables or comorbidities did not have the condition.” – how might this influence the results?

Page 11
Line 6 – Change “This” to “The”
Line 11 – write out full form of PPI in first usage

RESULTS
Page 11

	Line 39 – reword to "The mean age of patients was 81.9 years (SD 7.6) and 62.6% were female. Ethnicity records were available…"<br><br>Page 12<br>Line 13 – what does "slightly more prevalent" mean?<br>Were tests of significance performed? (Suggested in Table 1) If so, where are the quantitative results?<br><br>Page 17<br>Line 26 – what does "statistically significant trend of association" mean? This seems to be misleading given that 2/4 exposure categories for mirabegron were associated with increased dementia risk. The > 1095 group, while not statistically significant, is difficult to draw conclusions about given the low numbers.<br>Line 31 – What % of individuals on anticholinergic drugs had prior exposure to a different anticholinergic?<br><br>DISCUSSION<br><br>Page 20<br>Line 48 – ORs increased with all levels of mirabegron exposure except > 1095 (which had low sample size). It would be more accurate to state that the association between mirabegron and dementia risk was variable across exposure levels and may have been affected by prior anticholinergic bladder drug use.<br><br>Page 22<br>Line 36 – please also include a comparison to results of Matta 2022 which also examined the risk of dementia associated with the use of different bladder anticholinergic drugs in older adults. Unlike your study, they did not find an association between oxybutynin and tolterodine with incident dementia likely due to protopathic bias.<br><br>Page 23<br>Line 37 – please comment on differing affinities for brain M1 receptors as CNS penetration is not the only factor that impacts propensity for cognitive side effects<br>Line 56 – would take out "including the use of other non-drug interventions" since all the clinical guidelines cited (7-10) already emphasize the role of non-drug interventions<br><br>Page 24<br>Line 6 – take out "mirabegron is rarely prescribed as first-line treatment for older patients with overactive bladder, and" as this is redundant<br>Next steps<br>Based on results of Welk 2020 (27) and Matta 2022 along with what is known about the mechanism of mirabegron, I would suggest focusing on the need to incorporate findings of your study (and the others you mentioned) into the clinical guidelines for management of OAB. Rather than only emphasizing the need for more studies of mirabegron and dementia risk.<br><br>FIGURE 1<br>Forward-pointing arrows for exposure ascertainment and covariate measurement make it seem like these were measured prospectively. Consider removing arrowheads.<br><br>TABLE S2<br>Need new line before Alprazolam in bottom row

	References<br>1. Welk B, Richardson K, Panicker JN. The cognitive effect of anticholinergics for patients with overactive bladder. Nature Reviews Urology. 2021;18(11):686–700. doi:10.1038/s41585-021-00504-x<br>2. Matta R, Gomes T, Juurlink D, Jarvi K, Herschorn S, Nam RK. Receipt of overactive bladder drugs and incident dementia: A population-based case-control study. European Urology Focus. 2022;8(5):1433–40. doi:10.1016/j.euf.2021.10.009

**REVIEWER NAME**	Harron, Katie
**REVIEWER AFFILIATION**	
**REVIEWER CONFLICT OF INTEREST**	None
**DATE REVIEW RETURNED**	15-Feb-2024

**GENERAL COMMENTS**	Overall I think this is a clearly described study. There are the usual limitations of observational studies to be considered, most of which have been, and I have a few comments for clarification/consideration.<br><br>Abstract: The wording at the end of the results paragraph: “trend of association between dementia risk and exposure” isn’t clear without the context that is given in the main text. This should be reworded and you could consider describing this as a non-anticholinergic drug and using the term dose response instead of trend of association.<br><br>The main issue with this analysis, which I don’t think has been fully addressed, is the issue of indication bias. I don’t think we can tease apart the indication for the treatment (i.e. OAB) from the treatment itself, with the exception of the sensitivity analysis using mirabegron. This sensitivity analysis was based on small numbers and I can appreciate that there isn’t much you can say here - but it is interesting that the direction of effect seems to be the same as in the anticholinergic drug group. The sensitivity analysis was not powered to detect an effect. Are there another negative control that you could have used, with larger numbers? I think this could be discussed further, considering whether or not there could be a common cause of OAB and dementia that could explain the relationship. Finally it would be helpful to elaborate in the methods why you selected mirabegron as an additional exposure.<br><br>Was the issue of multiple testing considered? Table 4 presents a large number of odds ratios, some of which may be significant by chance.<br><br>How was matching on calendar time done? Was this the time of GP contact?<br><br>How exactly did you define treatment duration in days?<br><br>I am a little confused about the inclusion of the AEC score as a confounder within the multivariable analysis. As a confounder you are suggesting the AEC is associated with both the outcome of dementia (which seems appropriate given you described it as a measure of risk of cognitive decline) and the exposure of interest (i.e. the anticholinergic drug), which it is by design. But isn’t the AEC on the causal pathway between drug exposure and dementia? It

	isn't clear whether the AEC is only included for other drugs (i.e. not the exposure of interest) or not. The reasoning for including this variable as a confounder needs to be better justified.<br><br>IMD – was this based on the practice or the individual?<br><br>There are huge amounts of missing data on ethnicity and IMD (~50%). For ethnicity, Table 1 is confusing as it states there are 78,071 individuals with an ethnicity record, but that 1899 were classed as other/unknown. I would assume these should have been excluded from the table as with the other unknowns. It is difficult to infer the level of missingness according to case/control status – it would be helpful to include a line in the table for missing data on each of the variables (and BMI).<br><br>It is not clear whether or not dementia (i.e. the outcome) was included in the imputation model. What was the justification for imputing BMI but not smoking/alcohol variables and ethnicity?<br><br>How were the cut offs for drug exposure decided upon and why was the variable categorised rather than analysed continuously?<br><br>What were the characteristics of the 31 patients who had no eligible matched controls? i.e. why couldn't they be matched?<br><br>Table 4 suggests that associations were adjusted for other common anticholinergic medications. I don't think this is clear in the methods. Why just adjust for these three and not all of the ones you evaluated?<br><br>The footnote to table 3 is incomplete and the footnote to table 2 doesn't seem to be referenced in the table.<br><br>Table 3 – would be helpful to be clear that the cases are those with dementia.<br><br>This sentence should be re-worded: "Exposure to mirabegron was not associated with a statistically significant trend of association with dementia".

VERSION 1 – AUTHOR RESPONSE

<u>Reviewer #1 comments and responses</u>

Comment:

"Exposure Classification: I am unfamiliar with the literature on using Total Standardised Daily Dose (TSDD) for classifying anticholinergic exposure. It's essential to qualify this choice with appropriate references that link TSDD thresholds to clinical outcome risks. This is particularly important given the established relevance of the Drug Burden Index in pharmacological studies. (Nishtala PS, Allore H, Han L, Jamieson HA, Hilmer SN, Chyou TY. Impact of Anticholinergic Burden on Cognitive Performance: A Cohort Study of Community-Dwelling Older Adults. J Am Med Dir Assoc. 2020 Sep;21(9):1357-1358.e3. doi: 10.1016/j.jamda.2020.03.027. Epub 2020 May 10. PMID: 32402780; PMCID: PMC7971451). Clarifying why TSDD is a good measure of anticholinergic exposure would improve the paper's validity".

Response:

We thank the reviewer for this comment. Total Standardised Daily Dose (TSDD) has been used in numerous research studies to determine the risk of clinical outcomes associated with standardised exposure to drugs such as benzodiazepines (1, 2), combined central nervous system medication (3), as well as anticholinergic drugs (4, 5). Benzodiazepine use was shown to be significantly associated with incident loss of physical function in a 2002 study which used total standardised daily dose (number of days of use over the 6-month study period if used at the minimum effective dose) to represent the intensity of benzodiazepine use (1). Studies by Gray et al.(4) and Coupland et al.(5), found an increased risk of dementia associated with cumulative standardised exposure to anticholinergic medication.

Using the TSDD allowed us to quantify and standardise patients' cumulative exposure to different doses of the different bladder anticholinergic medication during the study period, using standardised conversion measures derived using the WHO recommended daily average maintenance dose of the drug(s).

Changes made: Although we had previously included the references by Gray et al.(4) and Coupland et al.(5) to our definition of Total Standardised Daily Dose (TSDD), we have now expanded this section to include more references as well as further clarify the benefit of using TSDD as our measure of bladder anticholinergic drug exposure. The subsection titled "Exposure variables" in the Methods section has now been revised with the following text:
"*Total standardised daily dose (TSDD) was used as the measure of patients' total cumulative exposure to each of the bladder anticholinergic drugs based on the method by Gray et al. (4) and Coupland et al. (5). TSDD has been used in numerous research studies to determine the risk of clinical outcomes associated with standardised exposure to drugs such as benzodiazepines (1, 2), combined central nervous system medication (3), as well as anticholinergic drugs (4, 5). The use of TSDD allowed us to quantify and standardise patients' cumulative exposure to different doses of the different medications during the study period using standardised conversion measures derived using the WHO defined daily dose (DDD)(6) (supplementary Table 1) which is the assumed average maintenance dose per day for a drug used for its main indication in adults*".

Comment:
"Strengths of the study: The methodology adopted by the authors to mitigate protopathic bias, notably excluding exposures three years before the index date, is commendable. This approach significantly strengthens the study's findings. The differential risk for delirium with anticholinergics(Nishtala PS, Chyou TY. Risk of delirium associated with antimuscarinics in older adults: A case-time-control study. Pharmacoepidemiol Drug Saf. 2022 Aug;31(8):883-891. doi: 10.1002/pds.5480. Epub 2022 May 25. PMID: 35587029; PMCID: PMC9545361.) has been studied previously. I agree with the novelty of this research in comparing the differential risk of dementia associated with various anticholinergics. Another noteworthy aspect is the use of mirabegron as a non-anticholinergic comparator for overactive bladder (OAB) drugs, enhancing the study's robustness".

Response:
We thank the reviewer for this positive assessment of the manuscript.

Comment:

"Addressing Confounders: Can the authors clarify if they accounted for the use of over-the-counter (OTC) antihistamines, which could potentially misclassify exposure? It should be noted as a limitation if this hasn't been considered".

Response:
In this study of routine electronic healthcare records from the clinical practice research datalink (CPRD), we accounted for all records of prescriptions for medications that had anticholinergic properties including commonly-used antihistamines such as chlorphenamine, cetirizine, loratadine and fexofenadine. While we know that antihistamines are also available over-the-counter, information on over-the-counter medication use is not captured in CPRD and is a limitation inherently associated with the use of routine electronic health records. We acknowledge the potential for residual confounding in our study and have now included this limitation in the manuscript. There is however no reason to believe a differential effect of this confounding between the case and control groups.

Changes made: The following text has now been included in the "strengths and weaknesses" section of the study:
"*Also, records of use of over-the-counter medication are not available in electronic health records. As such, there is the potential for residual confounding from patients' over-the-counter use of medication such as antihistamines which have some anticholinergic properties but could not be accounted for in the study analyses. There is however no reason to believe in a differential effect of this confounding between case and control groups*".

Comment:
"*Limitations: The manuscript would benefit from a more thorough discussion of the baseline differences between cases and non-cases, particularly in terms of BMI, alcohol consumption, smoking status, and depression. It's unclear whether covariate adjustment using propensity scores was considered to address these differences".*

Response:
We thank the reviewer for this comment. In the results section of the manuscript, we described the baseline characteristics of cases and controls. We also assessed for differences in the prevalence of comorbidities including mental health-related conditions such as anxiety, bipolar disorder and depression. We have now included more information in the results section, of the baseline differences between cases and controls in terms of BMI, alcohol consumption and smoking status.

Covariate adjustment using propensity scores was not used to address the baseline differences between cases and controls in our study. We adjusted for all patient sociodemographic characteristics, clinical variables and comorbidities by including these variables in the multivariable conditional logistic regression models (as described in the methods section and further included as text underneath the results tables).

Changes made: The following text has been added in the "Results" section of the study:
"*Mean BMI at baseline was slightly lower in cases than controls (25.7kg/m2 (SD 4.5) vs 26.5kg/m2 (SD 4.7) respectively). Prevalence of alcohol consumption (current alcohol drinking status) was lower in cases (47.7%) than controls (57.8%) but prevalence of current smoking was marginally higher at baseline in cases (7.8%) than controls (7.6%)*".

Comment:
"*Moreover, a deeper exploration of muscarinic receptor selectivity, beyond permeability to the blood-brain barrier (BBB), could provide a more nuanced understanding of the central anticholinergic*

adverse effects of antimuscarinics. The manuscript presents a somewhat oversimplified view of bladder anticholinergics' biological mechanisms and differential risks".

Response:
We thank the reviewer for this comment. This study is an observational epidemiological study in primary care so the overall insights are descriptive. In the discussion section, we had highlighted the possible mechanisms that may underlie our study finding of differential risks of dementia associated with bladder anticholinergic drugs. In light of the reviewer's comment, we have extensively revised the discussion section of the manuscript to provide deeper insights into the biological mechanisms underlying the differential adverse effects of antimuscarinics.

Changes made: We have included the following text in the discussion section (subsection titled "meaning of the study: possible explanations and implications"):

"*The therapeutic action of anticholinergic drugs in overactive bladder is exerted by blockade of the muscarinic M_3 receptors located on the bladder smooth muscle cells (7). Muscarinic M_1 and M_2 receptor subtypes in the brain play an important functional role in cognitive function (7). Interactions between anticholinergic drugs and especially the M_1 receptors in the brain have the potential to cause cognitive impairment, depending on muscarinic receptor binding profiles, lipophilicity and the ability to cross the blood-brain barrier (8). Small anticholinergic agents that have low molecular weight, are lipophilic and have neutral charge, such as oxybutynin, can easily cross the blood-brain barrier (9). Tolterodine, fesoterodine, solifenacin, and darifenacin are similar to oxybutynin in that they are also lipophilic tertiary amines which are partially unpolarized; however, unlike oxybutynin, their molecules are large (10). Trospium is a large, hydrophilic and positively charged molecules which does not readily cross the blood-brain barrier.*

Anticholinergic bladder drugs also differ in their affinity to the different muscarinic receptor subtypes (10). Darifenacin has the highest selectivity for the M_3 receptor over M_1 and M_2 subtypes, while solifenacin has only moderate selectivity of the M_3 receptor over M_1 and M_2 subtypes. Oxybutynin, fesoterodine, tolterodine, trospium and propiverine have been found to be non-selective for the M_3 receptor over the M_1 subtype".

Comment:
"*Overall Assessment: This is a well-conducted, large-scale pharmacoepidemiological study that contributes valuable insights to the existing evidence on the differential risks posed by anticholinergics concerning dementia*".

Response:
We thank the reviewer for the positive overall assessment of our study.

Reviewer #2 comments and responses

Comment:
"*The authors have conducted a very interesting study with mostly sound methodology*"

Response:
We thank the author for the positive assessment of the study

Comment:
"*1. Could the authors discuss the validity of the Mirabegron data in more depth, please. There appears to be unmeasured confounding with resulted in the significant risk in dementia. Mirabegron is used 2nd or 3rd line after a patient will have been taking for example Oxybutinin or Solifenacin for some time. Do the authors think the results are due to protopathic bias or confounding by indication?*"

Response:
We thank the reviewer for this comment on the validity of the mirabegron study findings. We acknowledge the possibility of unmeasured confounding which may occur with individuals who had prior OAB treatment with anticholinergic drugs, as well as possibility of confounding by indication whereby patients' might be prescribed mirabegron for OAB symptoms when they are thought to be at risk of cognitive decline. We have included more detail on this in the 'strengths and weaknesses' section of the manuscript.

Changes made: The following text have now been included in the "strengths and weaknesses section" of the manuscript:

"*Our study also assessed the risk of dementia associated with use of a non-anticholinergic drug, mirabegron. However as confirmed in our study where 86.2% of patients prescribed mirabegron had prior treatment with anticholinergic OAB medication, mirabegron is rarely prescribed as first-line treatment for OAB in UK clinical practice and so our study finding of increase in dementia risk with some categories of mirabegron exposure, may be due to unmeasured confounding from previous anticholinergic drug use. There is also the possibility of confounding by indication whereby patients with OAB symptoms are given first-line treatment with mirabegron instead of an anticholinergic OAB drug, when they are already thought to be at risk of cognitive impairment*".

Comment:
"*2. The drugs with insignificant findings happen to be the ones with very few numbers of patient taking them, hence also wide Confidence Intervals. Arguably, it is tricky to definitely rule out risk of dementia. These drugs may not be safer alternatives both from the risk of dementia aspect and other anticholinergic adverse effects"*.

Response:
The reviewer raises a fair point with this comment. We agree that the small number of patients within some of the anticholinergic drug exposure categories, does limit our statistical power in attaining precise measures of effect in these groups. In the manuscript, we have therefore not made any statements to definitely rule out the risk of dementia associated with these drugs. We state in the study findings, that "no significant increase in dementia risk was found with exposure to the other anticholinergic drugs…", and our study conclusion "emphasises the need for clinicians when treating overactive bladder in older adults, to take into account the possible long-term risks and consequences of the available treatment options and consider prescribing likely safer alternatives".

Reviewer #3 comments and responses

Comment:
I would like to thank the authors for this large, rigorous, observational study of the relationship between bladder anticholinergics and risk of dementia. I read the paper with great interest and have followed this line of inquiry for some time so I am familiar with the literature base. Given the pre-existing studies in this area I was surprised that the authors did not stratify the population by sex or by age of exposure > or < 80 years given the known differences in dementia risk in these populations. Is there a reason that this was not added, or commented on? I also think that the context of the discussion was too short and I would like to hear more about the gap that this particular analysis fills in relation to the previous studies on this topic.

Response:
We thank the reviewer for the positive assessment of the study.

We agree with the reviewer's comment that findings from previous research have demonstrated differences in dementia risk in the population of adults who were aged ≤ or >75 years (11) as well as in those aged < or > 80 years (5). Initially, we refrained from undertaking multiple subgroup analyses in this study to avoid potential type 1 errors related to multiple testing (12). Additionally, we were concerned about potentially compromising the study's statistical power and precision in estimating effect measures in subgroup analyses, especially given the small sample size in certain drug exposure categories. We have however now undertaken additional analyses to assess the association of bladder anticholinergic drugs with dementia risk in adults diagnosed with dementia

aged less than 80 years and those aged 80 years and over at diagnosis, and separately in males and females. As in the main analyses, associations with dementia were stronger with exposure to oxybutynin, solifenacin and tolterodine, however, these associations were stronger in the under 80s compared to those aged 80 years and over at diagnosis. In examining these associations by sex, a stronger association between dementia and exposure to any bladder anticholinergic drug was observed in males than females. In relation to the different OAB drugs, similar associations were found in males and females for oxybutynin while slightly stronger associations were observed in males for solifenacin and tolterodine. These analyses and the results/ discussion have been included in the manuscript.

Changes made: To reflect these additional analyses, we revised the manuscript sections below and included the text below:

Abstract: The adjusted odds ratio (AOR) for dementia associated with use of any bladder anticholinergic drug was 1.18 (95% CI 1.16 to 1.20*), and this was higher in males (1.22 [95% CI 1.18 to 1.26]) than females (1.16 [1.13-1.19])*.

Methods: "To ensure the study results were robust and generalisable, *subgroup analyses were done to assess associations in adults younger than 80 years and those aged 80 and over at time of dementia diagnosis/index date*, *associations in males and females separately,* and also within subgroup of patients prescribed mirabegron who had no prior exposure to any bladder anticholinergic drugs."

Results: *Sensitivity analysis assessed the association of bladder anticholinergic drugs with dementia risk in adults aged less than 80 years and those aged 80 years and over at time of dementia diagnosis/index date. Findings in both age-group categories were similar to the main analyses but associations between the bladder anticholinergics - oxybutynin, solifenacicn and tolerodine, with increased risk of dementia, were stronger in the under 80 age group than in adults aged 80 years and older (Supplementary Table S4a and Supplementary Table S4b). In separate subgroup analyses in males and females, the odds ratio for dementia associated with the use of any bladder anticholinergic drug was higher in males (AOR 1.22 [95% CI 1.18 to 1.26]) than females (AOR 1.16 [95% CI 1.13-1.19]). Associations were similar in males and females for oxybutynin, but slightly stronger in males than females for solifenacin and tolterodine. As in the main analyses, effect sizes for mirabegron were variables across exposure categories with no clear difference between males and females (Supplementary Table S5).*

Discussion: "*These associations were stronger in those who were aged less than 80 years at dementia diagnosis, than those aged 80 years and over. We found higher dementia risk associated with bladder anticholinergic drug use in males compared to females. While similar associations with dementia were observed in males and females for oxybutynin, slightly stronger associations were observed in males than females for solifenacin and tolterodine.*"

"Similar to the Canadian study (35), we found stronger associations between dementia and the use of any bladder anticholinergic drugs, in males compared to females. Unlike the study however, we further explored sex differences in dementia risk by type of bladder anticholinergic drug with findings of similar associations for oxybutynin, but slightly stronger associations in males for solifenacin and tolterodine. Although the reason for this differential risk in males and females in unclear, previous research suggests that males and females have different risk profiles for cognitive impairment and progression to dementia (40)."

"*Our finding that the bladder anticholinergics were associated with greater increase in dementia risk in those younger than 80 years at diagnosis, is similar to findings from a previous UK study which assessed dementia risk associated with anticholinergic drugs in people aged 55 years and over (5)*"

Reviewer #4 comments and responses

Comment:
GENERAL COMMENTS
- This is an important topic and the manuscript is generally well written.

Response:
We thank the reviewer for this positive comment and assessment of the study and manuscript

Comment:
- Reading the results, the main question is "Are these associations likely to be causal?" In favour of cause-effect are plausibility and temporality. But the lack of a compelling gradient in almost every analysis (adjusted ORs in Table 4) and the finding of associations where they are not expected (eg - for trospium, which should not cross the BBB, and for mirabegron, the notional comparator) seriously undermine the case for causation, especially in light of the modest effect sizes generated. There is no obvious remedy to this.

Response:
We thank the reviewer for this comment. Our study aimed to provide evidence of observed association and not causation, and we used methods for analysing matched case-control studies focused on utilising conditional logistic regression models that provide conditional and not causal estimates of the odds ratio. Therefore, we agree with the reviewer that causality cannot be directly attributed to the associations found in this study.

An 18% increase in dementia risk was found with the use of any OAB drug, yet the limited number of individuals exposed to some of certain medication categories resulted in the lack of a compelling

gradient in some analyses. Regarding the study findings with mirabegron, we have acknowledged the possibility of unmeasured confounding as well as indication bias with this analysis.

Comment:
- *The inclusion of younger cases (55-65) may adversely influence the signal:noise in this analysis because such patients are more likely to have a genetic cause of dementia (APP, PSEN1/2) rather than a drug-related component*.

Response:
We acknowledge the reviewer's viewpoint that younger cases aged 55 – 65 are more likely to have a genetic cause of dementia. However, in our epidemiological study on the association between OAB anticholinergic drugs and dementia risk, it is crucial to evaluate this association among all older adult age groups who may be prescribed OAB medications. As detailed above, we have now undertaken additional analyses to assess the association of bladder anticholinergic drugs with dementia risk in those aged <80 and those aged 80 and over, and the findings of a stronger association in the <80 group have been included in the manuscript.

Comment:
- *It's not clear whether controls were permitted to become cases at later points in time, as should be the case*

Response: The case-control matching for the study was done with replacement of controls, thereby allowing controls to be sampled several times including the opportunity for controls to become cases if there was a recorded diagnosis of dementia after the period of follow-up as controls.

Changes made: We have included the following revised text in the methods section:
"Each patient with dementia was matched with up to 5 controls without dementia, by age, sex, general practice, and calendar time using incidence density sampling *and allowing for replacement of controls*".

Comment:
- *We understand the reason for not including the 3 years prior to the index in the exposure assessment, but there might be value in including it in a sensitivity analysis*

Response:
We thank the reviewer for this comment.
Overactive bladder symptoms commonly occur in individuals with dementia (13) and previous studies on anticholinergic OAB drugs and dementia risk have been limited by including OAB drug prescriptions up to the time of dementia diagnosis (11) or shortly before (14, 15), potentially introducing protopathic bias, To address this bias, our study excluded exposure to these medications within the short-term period (3 years) prior to dementia diagnosis, aiming to determine the long term risk of dementia associated with the use of these drugs.
While we acknowledge the reviewer's suggestion to include records in the 3-year period before dementia diagnosis, this does not align with our study objectives and will introduce protopathic bias in the results. We will therefore be unable to offer any meaningful and reliable explanation for associations which may be observed with the inclusion of records within this time period.

SPECIFIC COMMENTS

- **ABSTRACT**

Page 3

Comment*: Line 28 – should be "TSDD" instead of "TSSD"*
Response: Line 28 has been revised. TSSD has been changed to TSDD.

Comment*: Line 48 – had to re-read as it wasn't clear what the 2nd AOR was referring to until the end of the sentence. Please reword*.
Response: Line 48 has been reworded as follows:
Among the different bladder anticholinergic drugs, dementia risk was found to be most significantly increased with use of oxybutynin (AOR 1.31 [95% CI 1.21 to 1.42] and 1.28 [1.15 to 1.43] for exposure of '366-1095' and '>1095' TSDDs respectively), solifenacin (AOR 1.18 [1.09 to 1.27] and 1.29 [1.19 to 1.39]) and tolterodine (AOR 1.27 [1.19 to 1.37] and 1.25 [1.17 to 1.34]) all for exposure of '366-1095' and '>1095' TSDDs respectively.

Page 4
Comment*: Line 5 – "trend of association" – it is not clear what this means (same wording used page 17 line 26) and I don't think this conclusion can be drawn based on the available results*
Response*:* This sentence has been changed from "There was no statistically significant trend of association between dementia risk and exposure to mirabegron" to "*The association between mirabegron and dementia was variable across the exposure categories and may be due to prior use of bladder anticholinergic drugs in these individuals*"

Comment*: Line 41 - median/IQR preferable to mean/SD*
Response*:* Line 41 (page 3): As recommended by the reviewer, we have now changed the value of age from mean (SD) to median (IQR) values. This has also been amended in the results section and included in table 1.

- **INTRODUCTION**

Page 5

Comment*: Line 13 – sounds like the "older population" refers to those > 20 years of age. Reword to: "Despite this, use of these drugs nearly doubled among older adults in England over the last 20 years (6)."*
Response*:* As suggested by the reviewer, this line has been reworded to improve clarity. We have reworded the text as: "*Despite this, use of these drugs nearly doubled in older adults in England over the last 20 years*".

Comment: *Line 18 – consider removing "debilitating" and "(in the absence of infection)"*
Response*:* This sentence been revised as suggested by the reviewer, with removal of the words *"debilitating" and "(in the absence of infection)".*

Comment: *Line 40 – anticholinergic drugs for OAB also differ in their affinity for M1 receptors in the brain. Please comment on this.*
Response*:* The statement which the reviewer refers to, was written to provide justification for variation in the mechanism of action and risk of cognitive side effects with the different bladder anticholinergic drugs. The text has now been rephrased as follows:

"*While anticholinergic drugs used in managing overactive bladder (OAB) have been associated with dementia risk as a group, individual medications vary in their ability to cross the blood-brain barrier under normal conditions (11, 12). Consequently, some anticholinergics may pose a higher risk than others. For instance, oxybutynin, a small molecule, readily crosses the blood-brain barrier and blocks M1 receptors. This action could contribute to the formation of neurofibrillary tangles and amyloid plaques associated with dementia (11). Additionally, higher doses of oxybutynin have been linked to greater memory problems (4, 11)*".

Comment*: Line 45 – potential contribution of oxybutynin to neurofibrillary tangles and amyloid plaques is not mentioned in reference 11. Where is this information from? Per Welk 2021 (see references), data on association between anticholinergic drugs and presence of plaques and tangles is mixed.*
Response*:* We thank the reviewer for highlighting our omission of the reference linking muscarinic blockade with amyloid plaques and neurofibrillary tangles. The relevant reference (16) has now been included in the manuscript.

Comment*: Line 58 – what does "more reassuring" mean? Was there no association with cognitive decline? If so, please state this explicitly.*
Response*:* In the manuscript, we state that "... higher doses of oxybutynin are associated with greater memory problems (4, 11). In contrast, a few clinical trials of short duration that have assessed the risk of cognitive decline associated with the use of solifenacin (13) and darifenacin (11) in older adults are more reassuring".

The use of the term "more reassuring" clearly suggests that unlike oxybutynin, solifenacin and darifenacin do not appear to negatively impact cognitive function. Following the reviewer's critique of the language used in the introduction, we have made revisions and altered the sentence to:

"*In contrast, a few clinical trials of short duration that have assessed the risk of cognitive decline associated with the use of solifenacin (13) and darifenacin (11) in older adults, have demonstrated no impairment of memory or other cognitive functions*".

Comment: Please include mechanism of mirabegron and rationale for why it has been included in the study.
Response: We have included the following paragraph and additional text in the introduction:
"*In addition to the use of anticholinergic drugs, overactive bladder can be effectively managed with mirabegron, a β3-adrenoceptor agonist. This medication works by inducing relaxation of the detrusor smooth muscle, suppressing detrusor overactivity, and enhancing bladder capacity (15). The NICE guidelines however, only recommend mirabegron as an alternative treatment for individuals in whom anticholinergic drugs are contraindicated or clinically ineffective (16)*".

"……Hence this study investigates the specific risk of dementia associated with the different anticholinergic drugs used for OAB, *as well as mirabegron*, in a very large, representative population of older individuals in England".

- **METHODS**

Page 7

Comment: *Line 29 – does this mean that controls could have a diagnosis of mild cognitive impairment or not otherwise specified cognitive decline?*

Response*:* We identified cases as individuals with a documented primary or secondary care diagnosis of dementia using an exhaustive list of codes for various types of dementia including vascular dementia, Alzheimer's dementia, other specified dementia types (for example Lewy body disease), and unspecified types of dementia. On the other hand, controls included individuals without any documented primary or secondary care diagnosis of dementia.
We already acknowledged that a limitation of the study which is inherent to the use of electronic health records, is the possibility of undiagnosed dementia or early cognitive impairment among individuals in the control group, and the consequent risk of misclassification which would shrink estimated effects towards the null.

Comment*: Line 42 – missing "as" before "this enabled exposure"*
Response*:* This has been amended in the manuscript

Page 8
Comment*: Line 24 – should be "TSDD" instead of "TSSD"*
Response: This has been amended in the manuscript

Comment: *In keeping with RECORD-PE item 7.1.g, please specify how participants with exposure to multiple OAB drugs were handled? From Table 4 it appears that they were included for each individual drug, meaning that participants may be counted > 1 time(s)*
Response: Our primary exposure of interest in this study was individuals' total cumulative exposure to the different OAB anticholinergic drugs. It is well-known that patients undergoing treatment for OAB are often exposed to multiple anticholinergic drugs. Therefore, we accounted for exposure to other commonly prescribed OAB medications in our multivariable conditional logistic regression analyses. This was initially stated under the respective tables, but it has now also been incorporated into the study methods.

Changes made: The following text has been included in the methods (statistical analyses) section of the manuscript:
"*As it is common in clinical practice for patients undergoing treatment for overactive bladder to be exposed to multiple anticholinergic drugs, we also accounted for exposure to other commonly prescribed OAB anticholinergic drugs in multivariable conditional logistic regression analyses*".

Page 9
Comment*: Please separate out variables known to increase risk of dementia and indications for other anticholinergic drug use*
Response: This section of the methods has been revised and the covariates are now listed by categories.

Comment*: Line 15 – "and" is missing before "type 2 diabetes"*
Response: We have included "and" between type 1 diabetes and type 2 diabetes.

Comment*: Line 26 – "We also accounted for patients' use of commonly prescribed overactive bladder anticholinergic drugs" – why is this included under the covariates section?*
Response: This has been removed from the list of covariates

Comment: *Line 38 – "…and academics with experience of research of anticholinergic drugs research" – typo*
Response: This has been amended and rephrased as "….*further discussion within the study team consisting of clinicians and academics with experience of research of anticholinergic drugs*"

Page 10
Comment: *Include use of Pearson's chi-squared test for baseline characteristics*
Response: The following text has been added to the section on statistical analyses: " *Pearson's chi-squared test was used to assess differences in categorical clinical variables between the groups*"

Comment: *Line 27 – "…assumptions were made that individuals with missing or unrecorded clinical variables or comorbidities did not have the condition." – how might this influence the results?*
Response: In research studies utilising electronic health records, such as the recently published QRisk4 algorithm development paper (17), it is often assumed that when there is no record of a binary clinical variable in a patient's record, then they do not have that particular condition. The manuscript has already recognised the limitations inherently associated with the use of electronic health records such as the possibility of non-recording or misdiagnosis of clinical information.

Page 11
Comment: *Line 6 – Change "This" to "The"*
Response: This has been changed

Comment: *Line 11 – write out full form of PPI in first usage*
Response: The term PPI was written in a section titled Patient and public involvement, which is the full form of PPI. As recommended by the reviewer, we have now also written this out in full form in the first mention of PPI in the text.

- **RESULTS**

Page 11

Comment: *Line 39 – reword to "The mean age of patients was 81.9 years (SD 7.6) and 62.6% were female. Ethnicity records were available…"*

Response: As recommended, this has been reworded. However, following this reviewer's earlier recommendation to change age from mean (SD) to median (IQR), this section of the results was amended to also reflect the change from mean (SD) to median (IQR). The new text reads:

"*The median age of patients was 83 years (IQR 77-87) and 62.6% were female. Ethnicity records were available for 42.4% of patients in the study and 95.1% of those with ethnicity records were White (Table 1)*".

Page 12

Comment: *Line 13 – what does "slightly more prevalent" mean?*
Response: We have amended this text to state: "*Asthma, hyperlipidaemia, and hypertension were more prevalent in controls than cases*".

Comment: *Were tests of significance performed? (Suggested in Table 1) If so, where are the quantitative results*?
Response: Tests of significance were performed, and the results have been included in Table 2.

Page 17

Comment: *Line 26 – what does "statistically significant trend of association" mean? This seems to be misleading given that 2/4 exposure categories for mirabegron were associated with increased dementia risk. The > 1095 group, while not statistically significant, is difficult to draw conclusions about given the low numbers*.

Response: This section has been amended and reworded as:
"*The risk of dementia associated with mirabegron was variable across different exposure categories, with an increased observed only with mirabegron exposure categories 91-365 TSDD and 366-1095 TSDD. This finding may be because 86.2% of individuals prescribed mirabegron (1,574 out of 1,826 patients) had prior exposure to bladder anticholinergic drugs. Additionally, the small number of individuals in the >1095 TSDD subgroup resulted in insufficient statistical power to detect precise measures of effect".*

Comment: *Line 31 – What % of individuals on anticholinergic drugs had prior exposure to a different anticholinergic?*

Response: Of the 78,787 individuals exposed to bladder anticholinergics, 77% (60,743) had prior exposure to at least one different bladder anticholinergic. The above has been included in the study results.

- **DISCUSSION**

Page 20

Comment: *Line 48 – ORs increased with all levels of mirabegron exposure except > 1095 (which had low sample size). It would be more accurate to state that the association between mirabegron and dementia risk was variable across exposure levels and may have been affected by prior anticholinergic bladder drug use*.

Response: ORs for dementia demonstrated an increase only in 2 of the mirabegron exposure categories (91-365 TSDD and 366-1095 TSDD), with no significant association in the 2 other categories (1-90 and >1095 TSDDs). The study discussion has been updated to reflect this, and has been reworded as follows:

"*Dementia risk varied among different exposure categories of mirabegron, a non-anticholinergic bladder drug, and this may be attributed to the prior use of bladder anticholinergic drugs in these individuals as well as the limited number of individuals in certain exposure categories*".

Page 22

Comment: *Line 36 – please also include a comparison to results of Matta 2022 which also examined the risk of dementia associated with the use of different bladder anticholinergic drugs in older adults. Unlike your study, they did not find an association between oxybutynin and tolterodine with incident dementia likely due to protopathic bias*.

Response: In the study discussion, we have now included a comparison of our study with that of Matta et al., and we have included the following text:

"*In the Canadian study by Matta et al., dementia risk was examined in 11,392 cases with dementia and 29,881 matched controls, comparing those who were exposed to different antimuscarinics with those exposed to mirabegron, even up to 6 months prior to diagnosis. Unlike our study, they did not observe a significant increase in dementia risk associated with oxybutynin and tolterodine. These findings are likely attributed to protopathic bias, with the study including treatments for prodromal symptoms of overactive bladder preceding the diagnosis of dementia.*"

Page 23

Comment: *Line 37 – please comment on differing affinities for brain M1 receptors as CNS penetration is not the only factor that impacts propensity for cognitive side effects*

Response: We have made significant changes to the manuscript discussion, incorporating more detail on the mode of action and varying affinity of the anticholinergic drugs to the muscarinic receptor subtypes. The following text has been included in the discussion:

"*The therapeutic action of anticholinergic drugs in overactive bladder is exerted by blockade of the muscarinic M3 receptors located on the bladder smooth muscle cells (34). Muscarinic M1 and M2 receptor subtypes in the brain play an important functional role in cognitive function (34). Interactions between anticholinergic drugs and especially the M1 receptors in the brain have the potential to cause cognitive impairment, depending on muscarinic receptor binding profiles, lipophilicity and the ability to cross the blood-brain barrier (35). Small anticholinergic agents that have low molecular weight, are lipophilic and have neutral charge, such as oxybutynin, can easily cross the blood-brain barrier (36). Tolterodine, fesoterodine, solifenacin, and darifenacin are similar to oxybutynin in that they are also lipophilic tertiary amines which are partially unpolarized; however, unlike oxybutynin, their molecules are large (37). Trospium is a large, hydrophilic and positively charged molecules which does not readily cross the blood-brain barrier.*

Anticholinergic bladder drugs also differ in their affinity to the muscarinic receptor subtypes (37). Darifenacin has the highest selectivity for the M3 receptor over M1 and M2 subtypes, while solifenacin has only moderate selectivity of the M3 receptor over M1 and M2 subtypes. Oxybutynin, fesoterodine, tolterodine, trospium and propiverine have been found to be non-selective for the M3 receptor over the M1 subtype".

Comment: *Line 56 – would take out "including the use of other non-drug interventions" since all the clinical guidelines cited (7-10) already emphasize the role of non-drug interventions*.

Response: Although we acknowledge the fact that clinical guidelines already suggest the use of non-drug interventions, we do not agree with the idea of excluding "including the use of non-drug interventions" from the aforementioned text. This text emphasizes the importance of cautious prescribing of oxybutynin, solifenacin, and tolterodine by clinicians, as well as the recommendation of safer alternatives, such as other non-drug interventions, for the treatment of overactive bladder in older adults. The manuscript's significant clinical and public health implication lies in highlighting the risks associated with commonly prescribed OAB anticholinergics and advocating for the consideration of potentially safer OAB treatments, including non-drug options.

Page 24

Comment: *Line 6 – take out “mirabegron is rarely prescribed as first-line treatment for older patients with overactive bladder, and” as this is redundant*.

Response: Although the reviewer expresses a subjective opinion that the aforementioned text is redundant, we respectfully disagree with their assessment. We firmly believe that the text offers valuable insight and context regarding the prescribing pattern observed in the study whereby most patients who were prescribed mirabegron had previously had treatments with OAB anticholinergic drugs. Furthermore, it elucidates why it was not possible to accurately evaluate the independent risk of dementia associated with cumulative exposure to mirabegron from our study.

- **Next steps**

Comment: *Based on results of Welk 2020 (27) and Matta 2022 along with what is known about the mechanism of mirabegron, I would suggest focusing on the need to incorporate findings of your study (and the others you mentioned) into the clinical guidelines for management of OAB. Rather than only emphasizing the need for more studies of mirabegron and dementia risk*.

Response: We believe that the study findings and clinical recommendations underscore the importance of safer prescribing practices for the anticholinergics which have been found to be associated with increased dementia risk, as well as the consideration of safer alternative medications. However, in the "Unanswered questions and future research" section, there is a call for more robust studies on the independent risk of dementia associated with exposure to mirabegron.

- **FIGURE 1**

Comment: *Forward-pointing arrows for exposure ascertainment and covariate measurement make it seem like these were measured prospectively. Consider removing arrowheads*.

Response: As requested by the reviewer, we have amended figure 1 by removing the forward-pointing arrowheads on the shapes depicting the timelines for “exposure ascertainment window” and “covariate measurement window”.

- **TABLE S2**

Comment: *Need new line before Alprazolam in bottom row*

Response: This has been amended

References

1. Welk B, Richardson K, Panicker JN. The cognitive effect of anticholinergics for patients with overactive bladder. Nature Reviews Urology. 2021;18(11):686–700. doi:10.1038/s41585-021-00504-x

2. Matta R, Gomes T, Juurlink D, Jarvi K, Herschorn S, Nam RK. Receipt of overactive bladder drugs and incident dementia: A population-based case-control study. European Urology Focus. 2022;8(5):1433–40. doi:10.1016/j.euf.2021.10.009

Reviewer #5 comments and responses

Comments to the Author: Overall I think this is a clearly described study. There are the usual limitations of observational studies to be considered, most of which have been, and I have a few comments for clarification/consideration.

Response: We thank the reviewer for this comment

Abstract**:**

Comment: *The wording at the end of the results paragraph: "trend of association between dementia risk and exposure" isn't clear without the context that is given in the main text. This should be reworded and you could consider describing this as a non-anticholinergic drug and using the term dose response instead of trend of association*.

Response: This section has been revised and reworded as follows:
"*The association between mirabegron, a non-anticholinergic drug, and dementia was variable across the exposure categories and may be due to prior use of bladder anticholinergic drugs in these individuals*".

Comment: *The main issue with this analysis, which I don't think has been fully addressed, is the issue of indication bias. I don't think we can tease apart the indication for the treatment (i.e. OAB) from the treatment itself, with the exception of the sensitivity analysis using mirabegron. This sensitivity analysis was based on small numbers and I can appreciate that there isn't much you can say here - but it is interesting that the direction of effect seems to be the same as in the anticholinergic drug group. The sensitivity analysis was not powered to detect an effect. Are there another negative control that you could have used, with larger numbers? I think this could be discussed further, considering whether or not there could be a common cause of OAB and dementia that could explain the relationship. Finally it would be helpful to elaborate in the methods why you selected mirabegron as an additional exposure.*

Response: We agree that indication bias (confounding by indication) is a possibility in this study whereby the risk of dementia is related to the indication for OAB treatments. However, due to the prolonged nature of prodromal symptoms (including OAB) prior to a diagnosis of dementia, we believe that protopathic bias is more relevant for our study. We made attempts to limit protopathic bias in the study by excluding records in the 3-year period before dementia diagnosis.

Mirabegron was included as a negative control in the study due to this medication having a different mechanism of action from the bladder anticholinergic drugs. We have expanded the introduction section to include text on the mechanism of action of mirabegron, prior to the justification for its inclusion in the study (for comparison) in the methods section. We have also acknowledged and extensively discussed our study limitation with the mirabegron analyses which is likely due to prior use of bladder anticholinergic drugs in these individuals.

Comment: *Was the issue of multiple testing considered? Table 4 presents a large number of odds ratios, some of which may be significant by chance*.

Response: As our research question assesses dementia risk associated with multiple exposure categories of OAB anticholinergics, we recognise the likelihood of type 1 errors due to multiple testing. Importantly, we acknowledge that the limited number of patients in certain drug exposure categories restricts our statistical power to obtain precise measures of effects within these groups. Nevertheless, the statistically significant association with oxybutynin, solifenacin and tolterodine holds scientific plausibility (18) and aligns with findings from a recent British cohort study of more harmful outcomes with oxybutynin and tolterodine compared to other bladder anticholinergic drugs when used in patients with dementia (19).

Comment: *How was matching on calendar time done? Was this the time of GP contact?*

Response: In matching by calendar-time, cases were matched with controls who were registered in the same general practice within the same time period as the case. To reflect this and avoid ambiguity, we have now revised the following text in the methods section:

"Each patient with dementia was matched with up to 5 controls without dementia, by age, sex, general practice, and calendar time (*same time period of follow-up*) using incidence density sampling and allowing for replacement of controls".

Comment: *How exactly did you define treatment duration in days?*

Response: Treatment duration in days was the patients' cumulative exposure (in days) to the different OAB medications during the study period. The methods section of the manuscript has been reflected as follows to clarify this:

"(b) Standardised exposure = standardised daily dose (per OAB drug type and per patient) multiplied by treatment duration (*cumulative exposure* (in days) *to the OAB drug during the study period*).

Comment: *I am a little confused about the inclusion of the AEC score as a confounder within the multivariable analysis. As a confounder you are suggesting the AEC is associated with both the outcome of dementia (which seems appropriate given you described it as a measure of risk of cognitive decline) and the exposure of interest (i.e. the anticholinergic drug), which it is by design. But isn't the AEC on the causal pathway between drug exposure and dementia? It isn't clear whether the AEC is only included for other drugs (i.e. not the exposure of interest) or not. The reasoning for including this variable as a confounder needs to be better justified*.

Response: In order to determine the independent risk of dementia associated with the different bladder anticholinergic drugs, our analyses took into account the patients' exposure to other anticholinergic drugs that are not used for the treatment of overactive bladder but which may increase the risk of dementia. In doing this, we classified the exposure to other anticholinergic drugs using a scale of 0 to 3 based on their known central anticholinergic burden and likelihood of being associated with cognitive impairment (20). It does state in the manuscript that the AEC score was derived for all non-bladder anticholinergic drugs that patients were exposed to during the study period.

Comment: *IMD – was this based on the practice or the individual?*

Response: We used patients' IMD (not practice IMD) in the study, and have clarified that in the methods section

Comment: *There are huge amounts of missing data on ethnicity and IMD (~50%). For ethnicity, Table 1 is confusing as it states there are 78,071 individuals with an ethnicity record, but that 1899 were classed as other/unknown. I would assume these should have been excluded from the table as with the other unknowns. It is difficult to infer the level of missingness according to case/control status – it would be helpful to include a line in the table for missing data on each of the variables (and BMI).*

Response: Table 1 has been revised as recommended. Ethnicity proportions are based on the total number of patients who had these in their records. We have included a footnote to explain that the proportion of individuals with ethnicity records was as a result of eligibility for linkage with Hospital Episode Statistics (HES). We have also included rows for missing data for IMD and BMI.

Comment: *It is not clear whether or not dementia (i.e. the outcome) was included in the imputation model. What was the justification for imputing BMI but not smoking/alcohol variables and ethnicity?*

Response: The outcome, dementia, was included in the multiple imputation model, and the manuscript has been updated to reflect this. Additionally, the references which suggest the inclusion of the outcome in the imputation model (21, 22) have been cited.

As stated in the manuscript, missing categorical variables such as ethnicity, smoking status and alcohol consumption, were addressed by the inclusion of a missing category indicator in the dataset. Our choice of multiple imputation over other methods for handling missing BMI records was based on the assumption that weight and BMI data are missing at random. In UK primary care, weight is recorded for only around a third of patients each year and repeated on average, every 2 years, and certain patient characteristics such as sex, age, alcohol consumption, smoking, and having several comorbidities, have been shown to be independently associated with an increased likelihood of weight recording (23). Multiple imputation is the recommended approach for handing missing weight or BMI records in epidemiological studies using primary care health records (24), where the practical analytical approach is to include in the imputation model, variable(s) that are predictive of the missing data (25). Previous research shows that the use of multiple imputation for missing weight records in primary care databases provided results comparable with population surveys(26).

Comment: *How were the cut offs for drug exposure decided upon and why was the variable categorised rather than analysed continuously?*

Response: The cumulative exposure to bladder anticholinergic drugs was classified based on the same categories used in previous studies of anticholinergic drugs (4, 5), which were determined by clinical interpretability and the exposure distribution seen in the primary care population of older adults. These references for the drug exposure categorisation have been added to the study manuscript.

Comment: *What were the characteristics of the 31 patients who had no eligible matched controls? i.e. why couldn't they be matched?*

Response: The matching criteria for selecting controls for cases included same age, sex, general practice and calendar-time (same time period as case). Inevitably, some older individuals with dementia who are registered in smaller general practices may not have eligible individuals of the same age and sex, registered at the same time. Out of 170,773 dementia patients, only 31 (0.018%) were excluded due to lack of eligible controls. Given that such a small proportion of dementia patients were excluded due to ineligible controls, we did not think it was appropriate or necessary to characterise and include these individuals in the study.

Comment: *Table 4 suggests that associations were adjusted for other common anticholinergic medications. I don't think this is clear in the methods. Why just adjust for these three and not all of the ones you evaluated?*

Response: The multivariable conditional logistic regression analyses had already taken into account patient variables such as sociodemographic characteristics, comorbidities, and the prescription of other non-bladder anticholinergic medications. Since the majority of these other OAB anticholinergic drugs were prescribed to less than 1% of the study population, there was a risk of overadjustment in the analyses, which may result in loss or imprecise measures of effect. In response to feedback from a previous reviewer, we have incorporated a statement in the methods highlighting the adjustment of the multivariable logistic model for exposure to other commonly prescribed OAB anticholinergic drugs.

Comment: *The footnote to table 3 is incomplete and the footnote to table 2 doesn't seem to be referenced in the table.*

Response: We thank the reviewer for highlighting these errors. We have amended the footnote to table 3 to include the full statement, and have also referenced the footnote to table 2 in the table.

Comment: *Table 3 – would be helpful to be clear that the cases are those with dementia*.

Response: The table title has been revised to provide clarification on the cases and controls being those with and without dementia respectively.

Comment: *This sentence should be re-worded: "Exposure to mirabegron was not associated with a statistically significant trend of association with dementia".*

Response: This sentence has been reworded as: *The risk of dementia associated with mirabegron was variable across different exposure categories.* There appeared to be an increase in dementia risk only with mirabegron exposure categories 91-365 TSDD and 366-1095 TSDD.

VERSION 2 – REVIEW

**REVIEWER NAME**	Harron, Katie
**REVIEWER AFFILIATION**	

**REVIEWER CONFLICT OF INTEREST**	None
**DATE REVIEW RETURNED**	02-Jul-2024

**GENERAL COMMENTS**	The authors have responded comprehensively to the extensive reviewer comments and I have nothing further to add.
